# A New Approach for the Synthesis of 2,3,4а,6,7,8а,9,10-Octaaza-4,8-dioxo-3,4,4a,7,8,8а,9,9a,10,10а-decahydroanthracene and High-Energy Performance Characterization of Its Dinitramide Salt

**DOI:** 10.3390/ma16237437

**Published:** 2023-11-29

**Authors:** Vera S. Glukhacheva, Sergey G. Il’yasov, Dmitri S. Il’yasov, Egor E. Zhukov, Ilia V. Eltsov, Andrey A. Nefedov

**Affiliations:** 1 Institute for Problems of Chemical and Energetic Technologies, Siberian Branch of the Russian Academy of Sciences (IPCET SB RAS), Biysk 659322, Russia; ilysow@ipcet.ru (S.G.I.); il.dmitriy82@mail.ru (D.S.I.); zhykov-e.e@yandex.ru (E.E.Z.); 2 Department of Natural Sciences, Novosibirsk State University, Novosibirsk 630090, Russia; eiv@fen.nsu.ru (I.V.E.); nefyodov@nioch.nsc.ru (A.A.N.); 3 Novosibirsk Institute of Organic Chemistry, Siberian Branch of the Russian Academy of Sciences (NIOCh SB RAS), Novosibirsk 630090, Russia

**Keywords:** dinitramide, anthracene hetero derivative, tricyclization reaction, ionic salts, burning rate inhibitor

## Abstract

A simple, one-pot regioselective method for the synthesis of a high-nitrogen tricycle, 2,3,4а,6,7,8а,9,10-octaaza-4,8-dioxo-3,4,4a,7,8,8а,9,9a,10,10а-decahydroanthracene, with a yield of 27% was developed on a starting urea basis as a result of studies focused on finding new, more efficient approaches to the synthesis of high-energy derivatives of dinitramic acid (DNA). This tricycle was further treated to furnish 2,3,4а,6,7,8а,9,10-octaaza-4,8-dioxo-3,4,4a,7,8,8а,9a,10а-octohydroanthracene-9,10-ion-bis(dinitramide). The resultant salt of dinitramic acid exhibited inhibitory properties towards the burning rate of pyrotechnic compositions, reducing it by 30%, and possessed good thermal stability due to a high decomposition temperature above 260 °C and a low sensitivity to mechanical stimuli. The structural features of the new tricycle-based dinitramide salt were characterized via 2D NMR spectroscopy and double-focusing sector mass spectrometry (DFS).

## 1. Introduction

The development of new high-energy materials around the world is closely associated with the derivatives of structurally different nitrogen heterocycles. These possess a high enthalpy of formation and enhanced density and are structurally rich in nitrogen that does need to be oxidized [1]. Moreover, energetic salts often have an advantage over non-ionogenic molecules. In particular, ionic compounds frequently have a higher density than atomically similar, non-ionogenic molecules because of the Coulomb forces influencing the formation of ordered and dense lattice structures in molecular assemblies [2,3,4,5,6]. Therefore, the combination of a heterocycle with an explosophoric group holds much promise for creating high-performance energy-rich structures [7,8].

Given the heightened interest in the development of novel, environmentally friendly and safe explosives with different uses (airbags, mining industry, fire protection, etc.), the dinitramide salts of linear and heterocyclic compounds, based on urea and diaminourea, warrant special attention [9,10,11,12]. The high content of oxygen and nitrogen and their high-energy properties allow these compounds to be used in gas-generating compositions or as composite explosives [13,14,15]. Even though dinitramide is quite an unstable compound, dinitramide anions are the most stable and energetically beneficial because negative charge is delocalized. That said, the salts of metals and amine bases are an ionic form of dinitramide [16,17,18,19,20].

**Figure 1:**
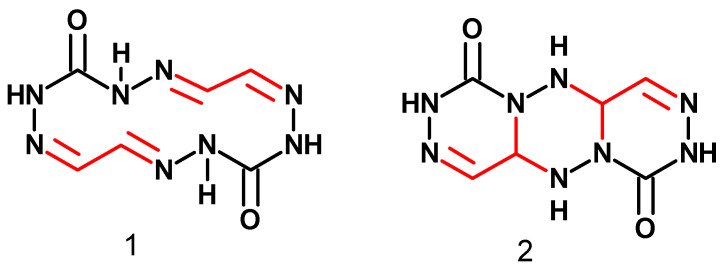
Macrocycle (**1**) and tricycle (**2**).

We previously reported the synthesis of new dinitramide salts, starting with carbohydrazide and a heterocyclic macrocycle (**1**), which inhibited properties with respect to the pyrotechnic composition burning rate [21,22,23] (Figure 1). In continuation of the research in this direction, we here propose investigating a structural analogue of compound **1** as the cation for the synthesis of new dinitramide salts. This analogue is a recently discovered tricycle, 2,3,4а,6,7,8а,9,10-octaaza-4,8-dioxo-3,4,4a,7,8,8а,9,9a,10,10а-decahydroanthracene (**2**), which is identical in elemental composition to compound 1 but distinct in constitution (Figure 1). 

Compound **2** was first synthesized via the intramolecular cyclodimerization of bis(nitrosemicarbazone)glyoxal in aqueous medium and represents a flat condensed system analogous to the reduced anthracene, contains ethylene, carbonyl and hydrazine moieties, and consists of three six-membered rings of 1,2,4-triazine and 1,2,4,5-tetrazine at the same time [24]. The presence of different functional groups in the structure of tricycle **2** implies its high reactivity. The functionalization of the tricyclic compound using various nitrating agents allows the diversity-oriented synthesis of both the acid salts and di- and tetranitro derivatives of the reduced anthracene. In contrast, the corresponding diazide was isolated via nitrosation [25]. However, the synthesis problems of the starting **2**, particularly the multistage process and low yields (at most 16% calculated on a starting urea basis), heavily hinder further research on expanding the compound’s practical application. 

Therefore, the present study aimed to optimize the synthetic procedure for tricycle **2**, develop a synthetic method for the corresponding dinitramide salt, and examine its explosive properties.

## 2. Materials and Methods

### 2.1. General Information

The ^1^H and ^13^C NMR spectra were acquired using Bruker Avance III 500 and Bruker AV-400 spectrometers (Bruker Corporation, Billerica, MA, USA), operating at frequencies of 500.03 MHz and 400.13 MHz for ^1^H NMR, and 125.73 MHz and 100.61 MHz for ^13^C NMR, respectively. The chemical shifts were referenced to DMSO-*d_6_* signals: 2.50 ppm for residual protons of *CH*D_2_ in ^1^H NMR spectra and 39.51 ppm for residual protons of CD_3_ in ^13^С NMR spectra. CDCl_3_ signals at 7.24 ppm were used as references for residual protons in ^1^H NMR spectra, as were signals at 77.16 ppm for ^13^С NMR spectra. The ^15^N NMR spectra were referenced to formamide as the external standard at δ(^15^N) = 112.5 ppm. Structural determination and signal assignment in the ^1^H,^13^C NMR spectra were performed using 2D heteronuclear techniques, including ^1^H,^13^C HMBC and ^1^H,^13^C HSQC.

UV absorption spectra were acquired using a Varian Cary 50 UV/Vis spectrophotometer (Varian, Belrose, Australia) in water, employing quartz cells (*l* = 0.5) at a temperature of 20 °C. Infrared spectra were recorded in KBr using an FT-801 Fourier spectrometer (Simex, Novosibirsk, Russia), with measurements performed in the range from 4000 to 500 cm^−1^. Elemental analysis was conducted using a CHNO FlashEATM 1112 analyzer (ThermoFisher Scientific, Waltham, MA, USA). The melting point was determined using a Böetius PHMK instrument (Veb Analytik, Dresden, Germany). Decomposition temperature measurements were carried out using TGA/SDTA 851e and DSC 822e thermal analyzers (Mettler Toledo, Zurich, Switzerland) under a nitrogen atmosphere, covering temperature ranges of 25–300 °C and 25–500 °C, respectively, with a heating rate of 10 °C/min. Data obtained from these experiments were digitized and processed using the STARe 11.0 thermal analysis software. 

Mass spectrometry and precise measurements of molecular weights were conducted using a Thermo Electron Double Focusing System (Thermo Electron Scientific Instruments Corporation, Madison, WI, USA). The samples, enclosed in metal vials, were introduced into the mass spectrometer via direct injection. When required, the sample vial could be heated within a temperature range from 25 to 360 °C. The mass spectrometer was utilized in the electron ionization mode, employing an electron energy of 70 eV. Precise measurements of ion masses were conducted using perfluorokerosene (PFK) standard lines as references.

### 2.2. Commercial Products Used

The following commercially available products were employed in this study:

- urea;
- a 40% aqueous solution of glyoxal;
- hydrazine hydrate;
- potassium perchlorate (ACS grade, technical specifications No. 6-09-3801-76, with a particle size ranging from 63 to 160 µm);
- aluminum (ASD-4 brand, technical specifications No. 48-5-226-87, with a particle size from 4 to 10 µm);
- potassium nitrate (ACS grade, compliant with GOST R 4217-77, with a particle size ranging from 63 to 160 µm); and
- zirconium (PCZr-1 brand, a powdered calciothermic Zr, technical specifications No. 48-4-234-84, with a particle size from 1 to 15 µm).

### 2.3. Synthetic Methods

1,2,4,5,8,9,11,12-Octaazacyclotetradeca-5,7,12,14-tetraene-3,10-dione (1)

(a) We added N,N′-dinitrourea (6.0 g, 0.04 mol) to water (100 mL) at 0–5 °C, and aqueous bis(hydrazone)glyoxal (3.44 g, 0.04 mol) was slowly poured into water (100 mL) with constant stirring. The whole was held for 0.5 h, and then heated to 60 °C. This temperature was held for 4 h. The precipitate was collected via filtration. Yield: 2.48 g (55.4%).
(b) A suspension of compound **3** (4.72 g, 0.02 mol) in water (100 mL) was heated to 70 °C and held at this temperature for 7 h, pH = 6. The whole was cooled, and the precipitate was collected via filtration and washed with water. Yield: 4.31 g (96.2%).

UV λ_1_ = 316 nm, λ_2_ = 200 nm. IR (cm^−1^): 3204, 3056, 1670, 1610, 1588, 1526, 1396, 1308, 1234, 1148, 934. NMR: ^1^H NMR (DMSO-d_6_ 400.13 MHz) δ_H_ (ppm): 7.363–7.400 (m, 2H, HC=N), 7.707 (m, 1H, NH), 7.842 (m, 1H, NH); ^13^C NMR (DMSO-d_6_ 100.61 MHz) δ_H_ (ppm): 136.06 (HC=N), 152.07 (C=O).

2,3,4а,6,7,8а,9,10-Octaaza-4,8-dioxo-3,4,4a,7,8,8а,9,9a,10,10а-decahydro-anthracene (2)

(a) We added N,N′-dinitrourea (12.0 g, 0.08 mol) to water (100 mL) at 0–5 °C, and aqueous bis(hydrazone)glyoxal (3.44 g, 0.04 mol) was slowly poured into water (100 mL) with constant stirring. The reaction mixture was held for 1 h, then heated to 70 °C and kept for 7 h at this temperature. The whole was cooled, and the precipitate was collected via filtration and washed with water. Yield: 2.39 g (53.3%).
(b) A suspension of compound 3 (4.72 g, 0.02 mol) in water (100 mL) was acidified with several drops of an acid (acetic or hydrochloric acid) to рН = 2, heated to 70 °C. and held at this temperature for 7 h. The whole was cooled, and the precipitate was collected via filtration and washed with water. Yield: 2.53 g (56.4%).

*Т_decomp_* = 253 °C. UV λ_1_ = 247 nm, λ_2_ = 204 nm, water. IR (cm^−1^): 3318, 3188, 3065, 3028, 2955, 2895, 1688, 1644, 1531, 1439, 1429, 1347, 1282, 1206, 1184, 1105, 1067, 984, 883, 830, 691. NMR: ^1^H NMR (DMSO-d_6_ 500.03 MHz) δ_H_ (ppm): 5.139–5.171 (dd, J = 10.4, 2.3; 2H, CH), 5.808–5.834 (m, 3 J = 10.7, 2H, NH), 6.693–6.698 (d, J = 2.3; 2H, HC=N), 10.348 (s, 2H, NH); ^13^C NMR (DMSO-d_6_ 100.61 MHz) δ_H_ (ppm): 69.55 (СН), 132.84 (C=N), 149.20 (C=O). HR-MS: Calcd for C_6_H_8_N_8_O_2_ [M]^+^ 224.0765; found *m*/*z* 224.0767. LR-MS, *m*/*z*, %: 224 (M^+^, 16), 113 (100), 112 (16), 98 (59), 56 (12), 44 (13), 43 (17), 42 (15), 40 (13).

Onium salt of [N,N′-bis(hydrazone)glyoxal]N,N′-dinitrourea (3)

We added N,N′-dinitrourea (3.0 g, 0.02 mol) to water (100 mL) at 0–5 °C, and aqueous bis(hydrazone)glyoxal (1.72 g, 0.02 mol) was slowly poured into water (100 mL) with constant stirring. The reaction mixture was kept at a temperature not above 22 °C for 4 h. The precipitate was collected via filtration and washed with water. Yield: 1.94 g (41.1%).

*Т_decomp_* = 235 °C. UV: λ_1_ = 213 nm, λ_2_ = 275 nm, water. IR (cm^−1^): 3209, 2989, 2898, 2819, 1692, 1605, 1548, 1501, 1421, 1209, 1190, 1080, 907, 880, 783. NMR: ^1^H NMR (DMSO-d_6_ 400.13 MHz) δ_H_ (ppm): 5.76 (s, 1H, CH), 6.96–7.22 (m, 3H, NH_3_^+^).

Calcd for С_6_H_16_N_16_O_10_: С, 15.25; Н, 3.39; N, 47.46. Found (%): С, 15.29; Н, 3.36; N, 47.50.

2,3,4а,6,7,8а,9,10-Octaaza-4,8-dioxo-3,4,4a,7,8,8а,9a,10а-octohydroanthracene-9,10-ion-bis(dinitramide) (4)

Portionwise, we slowly added compound **2** (2.24 g, 0.01 mol) with constant stirring to a solution of dinitramic acid (2.14 g, 0.02 mol) in water (50 mL) cooled to 0 °C. The whole was held for 1 h at a temperature not above 5 °C. The precipitate was collected via filtration and washed with water. Yield: 2.33 g (53.2%).

*Т_decomp_* = 265 °C. UV: λ_1_ = 206 nm, λ_2_ = 284 nm, water. IR (cm^−1^): 3427, 3317, 3187, 3065, 2885, 1691, 1644, 1599, 1530, 1439, 1282, 1205, 1184, 985, 883, 830. NMR: ^1^H NMR (DMSO-d_6_ 500.03 MHz) δ_H_ (ppm): 5.15 (d, 2H, CH), 6.68–6.69 (d, 2H, HC=N), 10.31 (s, 2H, NH); ^13^C NMR (DMSO-d_6_ 100.61 MHz) δ_H_ (ppm): 68.97 (СН), 132.28 (C=N), 148.65 (C=O). Calcd for С_6_H_10_N_14_O_10_: С, 16.44; Н, 2.28; N, 44.75. Found (%): С, 16.47; Н, 2.26; N, 44.78. 

### 2.4. Pyrotechnics

#### 2.4.1. Pyrotechnic Formulations

1. Potassium perchlorate (70%; <63 µm) and aluminum (30%; 4–10 µm);
2. Potassium nitrate (48%; 63–160 µm) and zirconium (52%; 1–15 µm).

Burning rate modifier: compound **4**. 

#### 2.4.2. Preparation of Ingredients and Fabrication of Pyrotechnic Compositions

All the components used herein were dried to constant weights. The powdered oxidizers were ground and screened using appropriate sieves.

The quantities of the substances being incorporated into the pyrotechnic compositions were 0.5%, 1.0% and 1.5%. The components were blended using an agate mortar.

#### 2.4.3. Measurements of Pyrotechnic Composition Burning Rate

Burning rate measurements were carried out under ambient pressure in air on cylindrical pyrotechnic charges of 10 mm in diameter, which were fabricated via pressing in a closed die. The pressing force was 20 kg/mm^2^. The burning rate was recorded using ionization detectors. The burning front passage time between the detectors was recorded on an AKTAKOM ASK-3107 oscillograph with a sampling frequency of 5 kHz. The burning was initiated by a spiral heating coil made of nichrome wire 0.5 mm wide. 

## 3. Results and Discussion

### 3.1. Synthetic Approaches

**Scheme 1:**
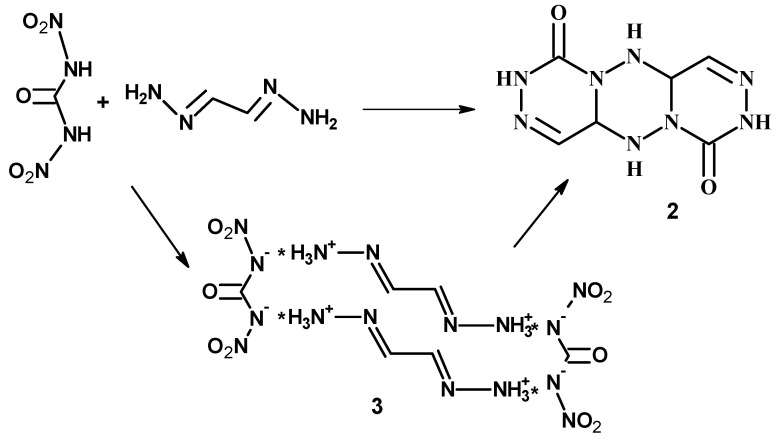
Synthesis of compounds **2** and **3**.

The synthesis of tricycle **2** (Scheme 1) was optimized towards reducing synthetic stages. For compound **2**, we considered the option of a direct interaction between bis(hydrazone)glyoxal and N,N′-dinitrourea (DNU) in aqueous medium upon heating under conditions identical to those of well-known synthetic procedures [24]. 

The decomposition of N,N′-dinitrourea in water refers to a second-order reaction; crystalline N,N′-dinitrourea in aqueous medium transitions to the aci-form [26]. It is well-known that the aci-form is a stronger acid that easily engages in salt-forming reactions with amines. As a result of the intermixing between the reactants, a ginger crystalline sediment of the bishydrazone salt of N,N′-dinitrourea (**3**) precipitated almost immediately in a quantitative manner, which is consistent with the reported data [26,27,28].

Compound **3** differs in the IR spectrum from the starting reaction products and has an onium structure, while the presence of a strong broad absorption band near 3209 cm^−1^ corresponds to the H-N bond vibrations in the NH_3_^+^ cation. The intense signal of a vibrational band near 1692 cm^−1^ is typical of the associated form of the amide С=О. The bending vibrations (δ_asym_) (H-N) show up at 1548 and 1501 cm^−1^. The absence of the absorption band near 3400–3300 cm^−1^ is suggestive of the N-Н bond being absent. Additionally, characteristic absorption bands are observed for asymmetric vibrations of the N-nitro group at 1605 cm^−1^ and for symmetric vibrations of the bond of the N-nitro group at 1289 cm^−1^.

**Figure 2:**
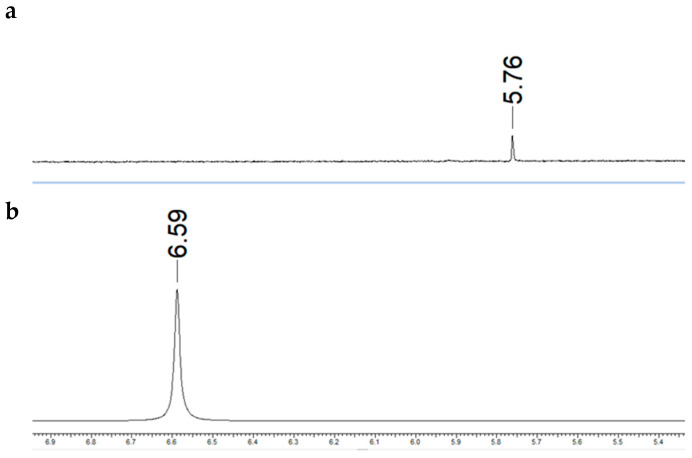
Comparison of ^1^H NMR spectra of (**a**) compound **3** and (**b**) bis(hydrazone)glyoxal in DMSO-d_6_.

It is seen in Figure 2 that the ^1^H NMR spectrum of the obtained sample of compound **3** has no singlet protons of the NH_2_ group of the starting bis(hydrazone)glyoxal at 6.59 ppm, while the characteristic signal for the double-bond proton shifts to 5.76 ppm due to the impact of the dinitrourea anion, in which case a characteristic triplet corresponding to the NH_3_^+^ cation protons shows up at 6.96–7.22 ppm [25]. The splitting of the signal into a few equivalent peaks suggests an interaction between the proton in question and the other non-equivalent protons. Considering the multiplicity rule, it can be affirmed that the number of protons found on the neighboring atoms of the nitramide proton of NH-NO_2_ equals 2, that is, the NH_2_ group of bis(hydrazone)glyoxal interacts with the nitramide group to form a NH_3_^+^ cation. The absence of free NH and NH_2_ protons is suggestive of the same ionization of the nitramide nitrogen atoms of DNU and of a complete neutralization of the H-N acid to form the aci-form of the nitro group with the NH_3_^+^ cation in the solution, which is consistent with the literature data [29]. 

**Scheme 2:**
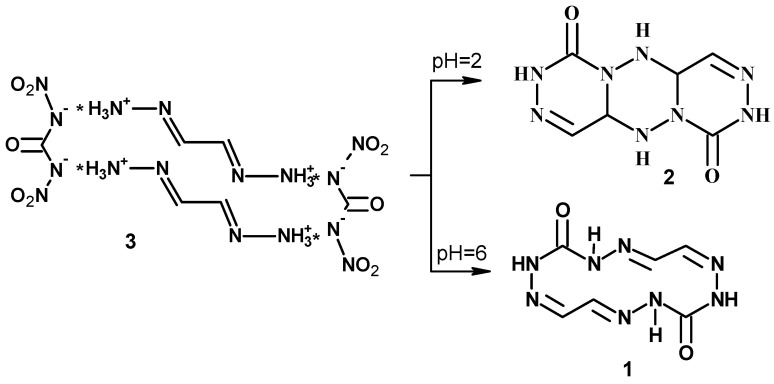
A synthetic scheme of compounds **1** and **2** from **3**.

Indirect proof of the structure of salt **3** can be attained via the further hydrolysis of the salt to furnish the respective cyclic product **1** or **2** (Scheme 2). Once compound **3** was isolated, the crystals of tricycle **2** began to settle down in the stock solution, as corroborated by X-ray diffraction analysis. 

The performed experiments in heating the aqueous solution of salt **3** at different pH values of the medium demonstrated that heating to 70 °C for 7 h at рН = 6 furnished monocycle **1** with up to 96% yield, while the acidification of the reaction mixture (with acetic or hydrochloric acid) to рН = 2 led to tricycle **2** being isolated with up to 56% yield. The resultant products were identified from the IR and NMR spectra and agreed with the literature data [24]. 

The process for the preparation of tricycle **2** was further optimized through a one-pot synthesis without isolating the intermediate salt **3**. The previous studies on the synthesis of tricycle **2** showed that the progress of intramolecular tricyclization requires an acidic medium that increases the activity of the acid proton, while a noticeable change in the medium acidity must be observed via the hydrolysis of DNU [30].

**Scheme 3:**
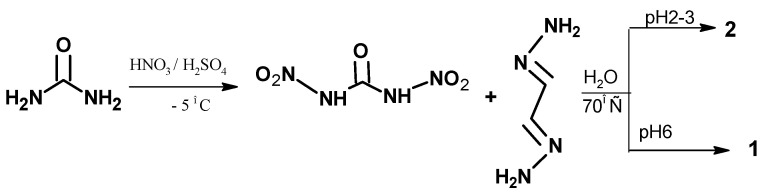
A synthetic scheme of **1** and **2** from urea.

**Figure 3:**
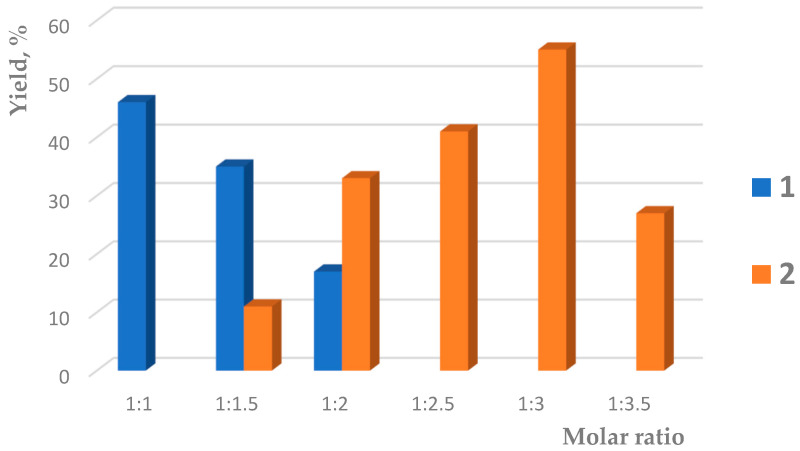
Yields of compounds **1** and **2** plotted against the molar ratio of the reactants at 70 °C for 7.

In order to confirm our assumption, we carried out a series of experiments on the interaction of aqueous bis(hydrazone)glyoxal with an excess of DNU in different molar ratios from 1:1 to 1:3 when heated to 70 °C for varied periods of time ranging from 3 to 7 h. The studies succeeded in isolating two types of sediments (Scheme 3). The reaction at an equimolar ratio of or in excess of DNU was found to proceed to furnish monocycle **1**. In a twofold or higher excess of DNU, the reaction yielded tricycle **2**. The results of the study into how the molar ratio, specifically excess DNU, influences the tricyclization reaction are illustrated in Figure 3. 

It follows from the obtained data that the threefold excess of DNU is an optimum molar ratio for the synthesis of compound **2**, while the equimolar ratio of 1:1 is optimum for the synthesis of compound **1**. 

That said, the reaction time also exerts an effect on the yield of the final product. The tricyclic compound **2** could be detected quantitatively only via 6 h of heating; until then, the recorded reaction products were salt **3** and monocycle **1**. 

Thus, we were able to elaborate a two-stage synthetic procedure for tricycle **2** from urea with a 27% yield calculated on a starting urea basis. 

**Scheme 4:**
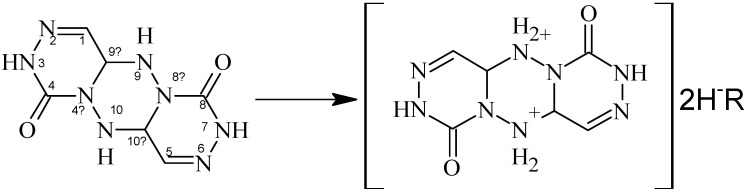
Protonation of compound **2**.

In the next step, we examined the salt-formation process of compound **2** with the dinitramide anion. The free protons on the nitrogen atoms at positions 3, 7 (the amide ones) and 9, 10 (the amine ones) were theoretically capable of being protonated with acid to generate the respective di- or tetranitro derivatives or salts. However, the previous studies showed that the basicity of the amide nitrogen was lowered by the impact of the electron-withdrawing carbonyl, and the amine protons of the central ring were involved in salt formation (Scheme 4) [25].

**Scheme 5:**
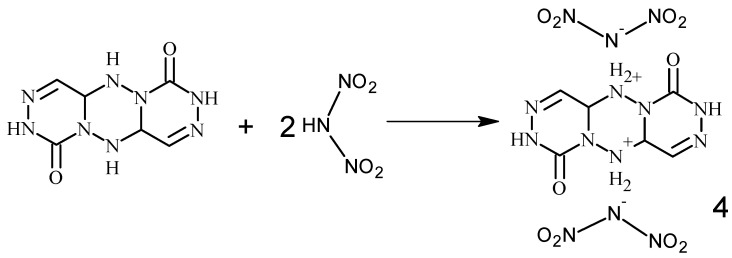
A synthetic scheme of **4** from **2** and **DNA**.

A similar reaction using dinitramic acid afforded 2,3,4а,6,7,8а,9,10-octaaza-4,8-dioxo-3,4,4a,7,8,8а,9a,10а-octohydroanthracene-9,10-ion-bis(dinitramide) (**4**), a high-melting crystalline compound in a 53% yield (Scheme 5). 

The IR spectrum of compound **4** retains stretching (ν) and bending (δ) vibrations of the N-H group of the tricycle at 3317, 3187 and 1644 cm^−1^, respectively. In this case, the characteristic stretching vibration of the amide carbonyl of the С=О group is observed to shift negligibly to 1691 cm^−1^. The stretching (ν) and bending (δ) vibrations of the С-Н bond show up at 3065 and 1439 cm^−1^, respectively, while out-of-plane vibrations show up at 688, 883 and 985 cm^−1^. In this case, characteristic intense absorption bands of the dinitramide group are observed to appear near 1599 and 1239 cm^−1^.

**Figure 4:**
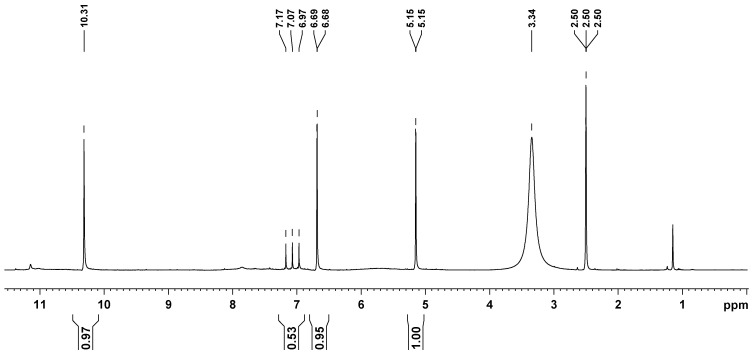
^1^H NMR spectrum of compound **4**.

**Figure 5:**
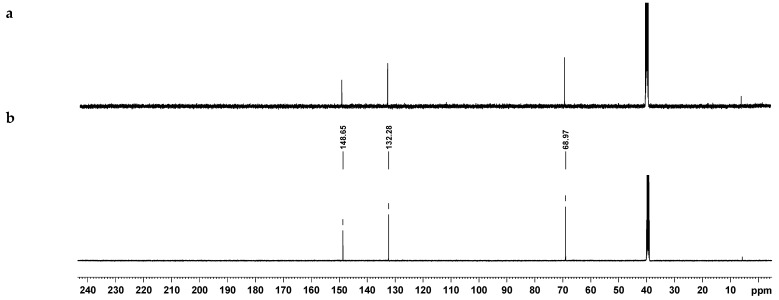
A comparison of ^13^С NMR spectra of (**a**) dinitrate salt [25] and (**b**) dinitramide salt **4** in DMSO-d_6_.

The ^1^H NMR spectrum of compound **4** (Figure 4) is analogous to the previously reported ones [25]; signals are observed from the solvent (DMSO, 2.50 ppm), water (3.34 ppm), ammonium cation (1:1:1—a triplet, 7.07 ppm), just as we observe three signals from the cation corresponding to hydrogen atoms of the CH, =CH and NH groups at 5.15, 6.69 and 10.31 ppm. The mutual ratio of the integral intensities is 1:1:1 and matches the assumed structure. The analyzed ^13^С{^1^H} NMR spectra (Figure 5). also show that the signals from compound **4** and the previously described salt of the tricycle fully coincide at 69, 132 and 149 ppm.

**Figure 6:**
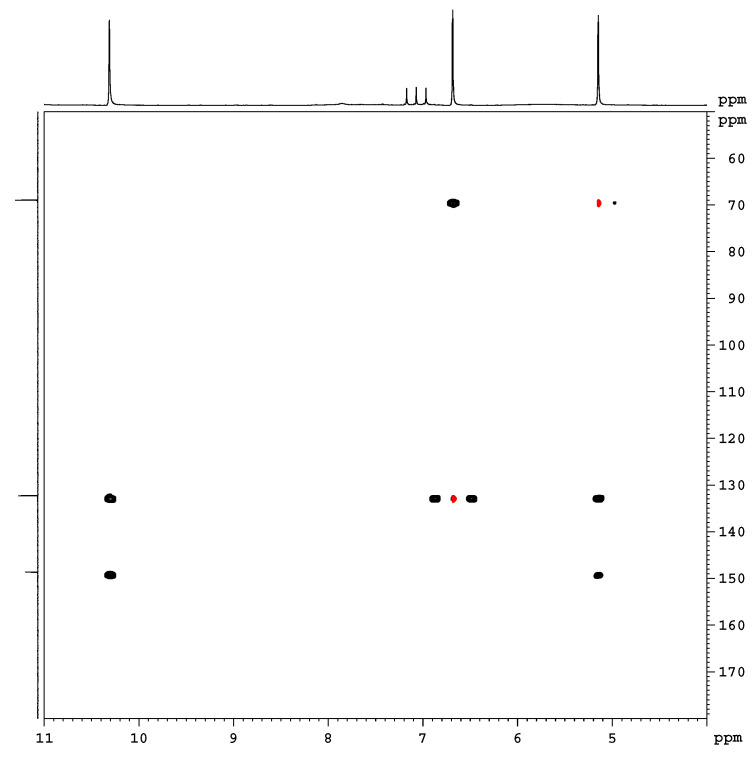
^1^H,^13^C HSQC (red) and ^1^H,^13^C HMBC (black) spectra of compound **4**.

**Figure 7:**
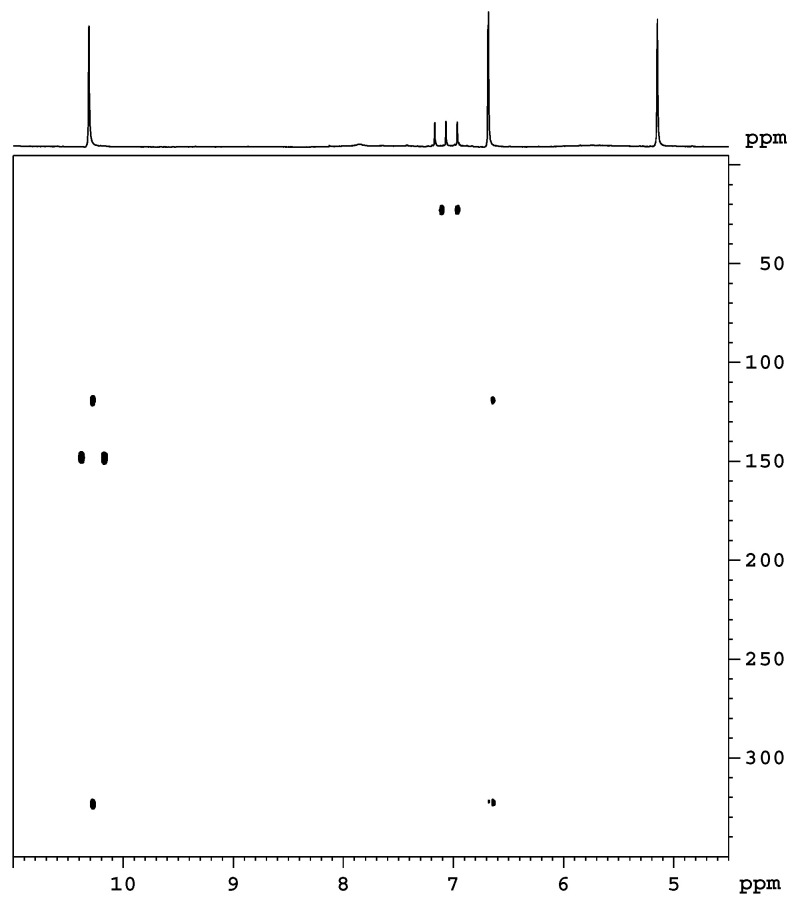
^1^H,^15^N HMBC spectrum of compound **4**.

The structural analysis of the cation framework performed on the samples of compound **4** by 2D ^1^H,^13^C HSQC, ^1^H,^13^C HMBC and ^1^H,^15^N HMBC showed it to be identical to the previously studied tricycle **2** (Figure 6 and Figure 7). 

The signals from the NH groups belonging to the central ring of the cation did not show up in either the ^1^H NMR spectrum or in the ^1^H,^15^N HMBC spectrum. This might be suggestive of these hydrogen atoms being involved in a chemical exchange with water found in the sample or of the nitrogen atom being partially protonated with the acid included in the salt composition. The nearly full identity of chemical shifts of the other atoms, as compared to tricycle **2**, indicates that no substantial ionization of the substance occurred under the conditions used. 

**Figure 8:**
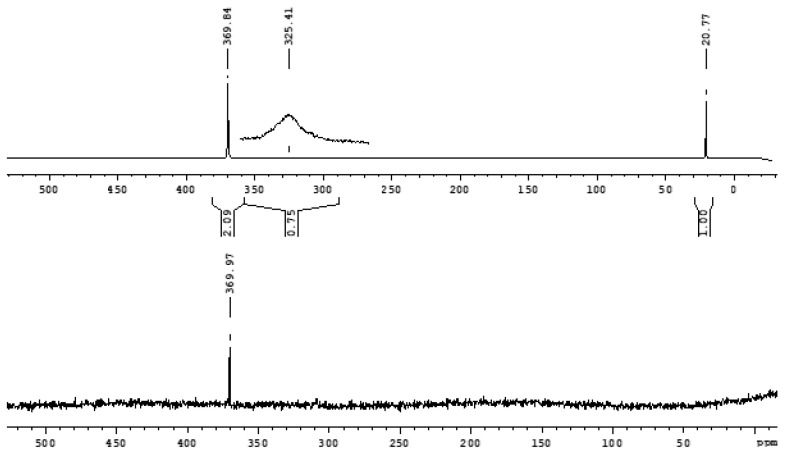
^14^N NMR spectra of the samples of **4** and ADN (from bottom to top).

That said, the obtained picture of the spectra coincides with that of the previously reported compound **2** [24]; therefore, an additional analysis of the counter-cation was performed using 1D ^14^N NMR spectroscopy. Due to the concentration of the substance being lower, we could detect only one signal near 370 ppm, corresponding in its position to the nitrogen atom of the nitro group of the ammonium dinitramide (ADN) sample (Figure 8). 

It can thus be said that compound **4** is fully consistent with the assumed structure. The cation portion represents tricycle **2**, in which the NH groups of the central moiety of the tricycle are involved in the chemical exchange and do not show up in the spectrum. The anion portion is also quite consistent with the anticipated results, corroborating the formation of the dinitramide salt of tricycle **4**.

**Scheme 6:**
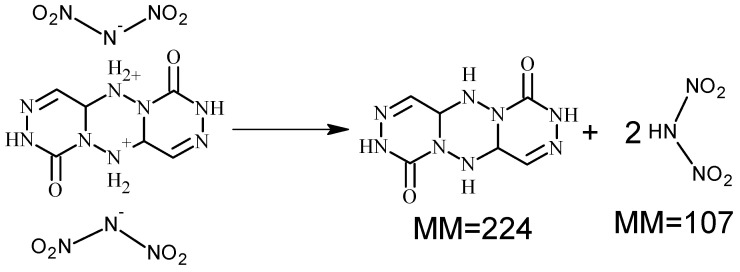
An assumed disassociation of compound **4**.

Mass spectrometry of compound **4** demonstrated the ion of salt **4** (*m*/*z* = 438) to be absent in the spectrum, which was expected because disassociate salts in mass spectrometry analyses and as the disassociation was expected to follow Scheme 6 in this case (see Supplementary Materials). 

The molecular ion with *m*/*z* = 224, the line of tricycle **2** and its related ions with *m*/*z* = 113, *m*/*z* = 112, *m*/*z* = 98, etc., were further measured. There was no molecular ion of ammonium dinitramide (*m*/*z* = 107), but there were nitro group ions with *m*/*z* = 46 (NO_2_) and *m*/*z* = 30 (NO). It seems that disproportionation/decomposition of dinitramide occurred under the experimental mass spectrometry conditions because intramolecular redox products with *m*/*z* = 18 (H_2_O) and *m*/*z* = 28 (N_2_) were observed.

### 3.2. Physical and High-Energy Properties

The thermogravimetric (TGA) and differential thermal analyses (DTA) in the polythermal mode under nitrogen conditions at a heating rate of 10 °C/min showed the good thermal stability of the resultant compound **4**. A 2.6% weight loss of the sample was found to occur in a temperature range of up to ≈100 °C. This was likely due to the evaporated hydrated water and coincides with similar effects for the other dinitramide salts [21,22,23]. In the DSC spectrum, a prominent decomposition peak is detected at 265 °C, accompanied by a heat release of 437 J/g. Additionally, a significant weight loss of 68.15% is observed, indicating a pronounced exothermic effect. The solid residue was 29.22%. According to existing literature, the decomposition peak at 199 °C in compound **4** is attributed to the decomposition of the constituent part of the dinitramide anion. On the other hand, the decomposition peak observed at 265 °C corresponds to the constituent part of tricycle **2**.

Synthesized salt **4** is a crystalline substance that is almost insoluble in water and alcohol and which explodes when heated; the physicochemical properties and sensitivities to impact and friction compared to starting ADN are summarized in Table 1. 

It is seen in Table 1 that the synthesized salts **3** and **4** are of interest as safe high-energy compounds because they have reduced sensitivity to mechanical stimuli and enhanced thermal stability with negligibly reduced density when compared to ADN.

**Figure 9:**
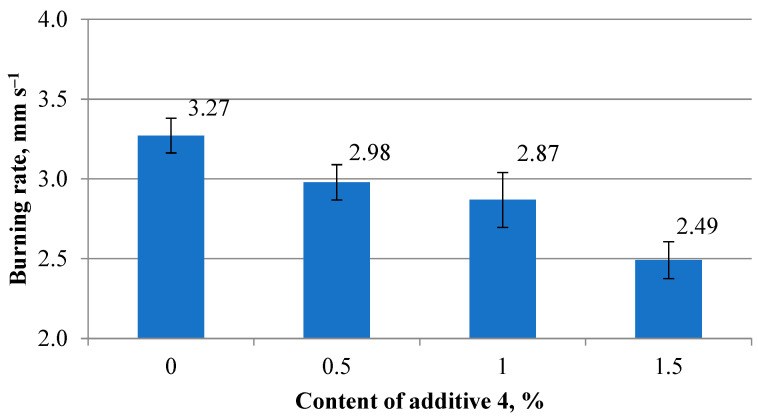
The effect of compound **4** on the burning rate of the KClO_4_/Al pyrotechnic composition at a pyrotechnic charge density of 2.25–2.35 g/cm^3^.

**Figure 10:**
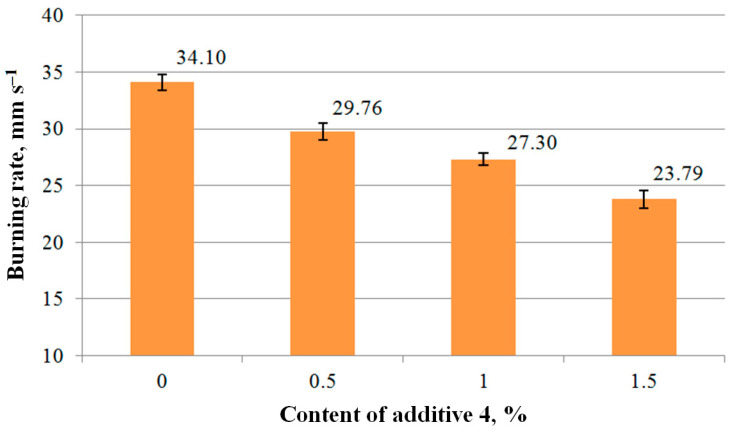
The effect of compound **4** on the burning rate of the KNO_3_/Zr pyrotechnic composition at a pyrotechnic charge density of 2.75–2.95 g/cm^3^.

To evaluate the catalytic or inhibitory effect of compound **4** on the burning rate of pyrotechnic compositions, we performed comparative tests on model compositions (well-known pyrotechnic formulations exhibiting both low and high burning rates (*u*)) via the reported procedure [36]. The test results are shown in Figure 9 and Figure 10. 

The low-rate formulation represented mixed 70/30% potassium perchlorate/aluminum, with a burning rate of 3.27 mm/s without additives. 

The high-rate formulation was a mixture of 48% potassium nitrate and 52% zirconium and had a 34.10 mm/s burning rate without using additives.

The obtained findings demonstrate that the burning rate decreases in both cases and is in direct relationship with an increase in the content of compound **4** in the KClO_4_/Al and KNO_3_/Zr systems. Table 2 presents comparative data on the relationship between the inhibitory effect and the additive content.

The results outlined in Table 2 demonstrate that compound **4** exhibits a pronounced inhibitory effect ranging from 5% to 30.23%. That said, the high-rate formulation is more noticeably susceptible to the effect of additive **4**.

## 4. Conclusions

1. In the current study, a novel regioselective approach for synthesizing 2,3,4a,6,7,8a,9,10-octaaza-4,8-dioxo-3,4,4a,7,8,8a,9,9a,10,10a-decahydroanthracene, which is a promising high-nitrogen tricycle with a high nitrogen content, was discovered.
2. A never-before-seen structure of the corresponding bis-dinitramide salt was synthesized by reacting dinitramic acid with the tricycle followed by protonation of the H-N protons of the central ring.
3. The tricycle-based dinitramide salt is appealing as a safe high-energy material that has a low sensitivity to mechanical stimuli, enhanced thermal stability, and acts as a burning rate inhibitor of pyrotechnic compositions.

## Figures and Tables

**Table 1:** Physicochemical properties of compounds **3** and **4** compared to ADN.

Comp.	Oxygen Balance	Density, g/cm^3^	Т_dec._, °C	Sensitivity, Lower Limit	
				Impact (10 kg), mm a per GOST R 4545-88 in Tester No. 2	Friction, kgf/cm^2^ as per GOST R 50835-95
**2**	–99.92	1.5321	252 [24]	-	-
**3**	–33.88	1.4692	235	<50	2400
**4**	–11.59	1.6506	265	70	3200
ADN [30,31,32,33]	25.80	1.8120	91–94	90	3000

Note: The lower impact sensitivity limit is a minimum drop height at which explosion occurred in at least one of 25 tests and was measured using a drop weight impact testing machine by dropping a weight. Triggering was detected from a sound effect and/or soot formation on the rollers [34]. The lower friction sensitivity limit is a maximum applied pressure on a highly explosive charge placed between steel planes, under which no single explosion occurred in 25 tests upon a shock displacement of one plane relative to the other and was measured using a K-44-3 friction testing machine [35].

**Table 2:** The burning rate (∇*u*) depending on the content of **4** in the formulations.

Additive	∇*u* (KClO_4_/Al), %	∇*u* (Zr/KNO_3_), %
0.5%	−8.87	−12.73
1.0%	−12.23	−19.94
1.5%	−23.85	−30.23

## Data Availability

Data are contained within the article and Supplementary Materials.

## Supplementary Materials

The following supporting information can be downloaded at: www.mdpi.com/ma16237437/s1, containing IR spectra, DSC and TGA data, and burning rate test results for compounds **2**, **3** and **4**.

## References

[1] Bottaro C., Schmitt R.J., Penwell P.E., Ross D.S. (1993). Method of Forming Dinitramide Salts. U.S. Patent.

[2] Drake G., Hawkins T., Brand A., Hall L., Mckay M., Vij A., Ismail I. (2003). Energetic low-melting salts of simple heterocycles. Propellants Explos. Pyrotech..

[3] Schaller U., Lang J., Gettwert V., Hurttlen J., Weiser V., Keicher T. (2022). 4-Amino-1-Butyl-1,2,4-Triazolium Dinitramide—Synthesis, Characterization and Combustion of a Low-Temperature Dinitramide-Based Energetic Ionic Liquid (EIL). Propellants Explos. Pyrotech..

[4] Kumar P. (2020). An overview over dinitramide anion and compounds based on it. Indian Chem. Eng..

[5] Klapoetke T.M., Martin F.A., Mayr N.T., Stierstorfer J. (2010). Synthesis and characterization of 3,5-diamino-1,2,4-triazolium dinitramide. ZAAC.

[6] Klapoetke T.M., Stierstorfer J. (2009). Azidoformamidinium and 5-aminotetrazolium dinitramide-two highly energetic isomers with a balanced oxygen content. Dalton Trans..

[7] Luk’yanov O.A., Parakhin V.V., Shlykova N.I., Dmitrienko A.O., Melnikova E.K., Kon’Kova T.S., Monogarov K.A., Meerov D.B. (2020). Energetic: N-azidomethyl derivatives of polynitro hexaazaisowurtzitanes series: CL-20 analogues having the highest enthalpy. New J. Chem..

[8] Vinogradov D.B., Bulatov P.V., Petrov E.Y., Tartakovsky V.A. (2020). Heterocyclization of amino alcohols into saturated cyclic quaternary ammonium salts. Mendeleev Commun..

[9] Jianrong R., Dong C., Guijuan F., Ying X., Zhenqi Z., Yanwu Y., Hongzhen L. (2019). Synthesis, characterization and properties of a novel energetic ionic salt: Dicarbohydrazide bis[3-(5-nitroimino-1,2,4-triazole)]. New J. Chem..

[10] Fischer N., Klapotke T.M., Stierstorfer J. (2011). Explosives based on diaminourea. Propellants Explos. Pyrotech.

[11] Fischer N., Klapotke T.M., Reymann M., Stierstorfer J. (2013). Nitrogen-rich salts of 1H,1′H-5,5′-bitetrazole-1,1′-diol: Energetic materials with high thermal stability. Eur. J. Inorg. Chem..

[12] Oxley J.C., Smith J.L., Vadlamannati S., Brown A.C., Zhang G., Swanson D.S., Canino J. (2013). Synthesis and characterization of urea nitrate and nitrourea. Propellants Explos. Pyrotech..

[13] Venkatachalam S., Gopalakrishnan S., Kovoor N.N. (2004). An Overview on the Synthetic Routes and Properties of Ammonium Dinitramide (ADN) and other Dinitramide Salts. Propellants Explos. Pyrotech..

[14] Sims S., Fischer S., Tagliabue C. (2022). ADN solid propellants with high burning rates as booster material for hypersonic applications. Propellants Explos. Pyrotech..

[15] Larsson A., Wingborg N., Hall J. (2011). Chapter 7. Green Propellants Based on Ammonium Dinitramide (ADN). Advances in Spacecraft Technologies.

[16] Lukyanov O.A., Gorclik V.P., Tartakovsky V.A. (1994). Dinitramide and its Salts 1. Russ. Chem. Bull..

[17] Lukyanov O.A., Anikin O.V., Gorclik V.P., Tartakovsky V.A. (1994). Dinitramide and its salts 3. Russ. Chem. Bull..

[18] Alavia S., Thompson D.L. (2003). Decomposition pathways of dinitramic acid and the dinitramide ion. J. Chem. Phys..

[19] Ghee A.H., Santhosh G. (2007). Advances in Energetic Dinitramides: An Emerging Class of Inorganic Oxidizes.

[20] Ermolin N.E., Fomin V.M. (2016). On the mechanism of thermal decomposition of ammonium dinitramide (review). Combust. Explos. Shock Waves..

[21] Il’yasov S.G., Glukhacheva V.S., Il’yasov D.S., Zhukov E.E., Eltsov I.V., Gatilov Y.V. (2022). A novel energetic nickel coordination compound based on carbohydrazide and dinitramide. Mendeleev Commun..

[22] Il’yasov S.G., Glukhacheva V.S., Il’yasov D.S., Zhukov E.E. (2022). Macrocycle as a “Container” for Dinitramide Salts. Materials.

[23] Il’yasov S.G., Glukhacheva V.S., Il’yasov D.S., Zhukov E.E., Eltsov I.V., Nefedov A.A., Gatilov Y.V. (2023). Adducts of the zinc salt of dinitramic acid. Materials.

[24] Glukhacheva V.S., Il’yasov S.G., Obraztsov A.A., Gatilov Y.V., Eltsov I.V. (2018). A New Synthetic Route to Heteroanthracenes. Eur. J. Org. Chem..

[25] l’yasov S.G., Glukhacheva V.S., Yermoshina V.A., Eltsov I.V. (2019). Synthesis of nitro-octaaza derivatives of reduced anthracene. ZAAC.

[26] Il’yasov S.G., Lobanova A.A., Bagryanskaya I.Y., Rybalova T.V., Gatilov Y.V. (2009). Physicochemical studies of the structure of n,n′-dinitrourea and its salts. J. Struct. Chem..

[27] Ye C., Gao H., Twamley B., Shreeve J.M. (2008). Dense energetic salts of N,N-dinitrourea (DNU). New J. Chem..

[28] Guo Y., Tao G.H., Joo Y.H., Wang R., Twamley B., Parrish D.A., Shreeve J.M. (2009). Impact insensitive dianionic dinitrourea salts: The CN_4_O_5_^2−^ anion paired with nitrogen-rich cations. Energy Fuels..

[29] Lobanova A.A., Sataev R.R., Popov N.I., Ilyasov S.G. (2000). Chemistry of urea nitro derivatives: I. synthesis of N,N-dinitrourea. Russ. J. Org. Chem..

[30] Zhang Y., Ma B., Jia X., Hou C. (2023). Prediction of ethanol-mediated growth morphology of ammonium dinitramide/pyrazine-1,4-dioxide cocrystal at different temperatures. Molecules.

[31] Chen F., Xuan C., Lu Q., Xiao L., Yang J., Hu Y., Zhang G., Wang Y., Zhao F., Hao G. (2023). A review on the high energy oxidizer ammonium dinitramide: Its synthesis, thermal decomposition, hygroscopicity, and application in energetic materials. Def. Technol..

[32] Nagamachi M.Y., Oliveira J.I.S., Kawamoto A.M., Dutra R.D.C.L. (2009). ADN—The new oxidizer around the corner for an environmentally friendly smokeless propellant. J. Aerosp. Technol. Manag..

[33] Bottaro J.C. (1996). Recent advances in explosives and solid propellants. Chem. Ind..

[34] (1988). High Explosives. Test Methods for Impact Sensitivity.

[35] (1997). High Explosives. Test Methods for Friction Sensitivity upon Shock Displacement.

[36] Belyaev A.F., Bobolev V.K., Korotkov A.I., Sulimov A.A., Chuiko S.V. (1973). Transition of Combustion of Condensed Systems into Explosion.

