# Supplementary material for: A New Approach for the Synthesis of 2,3,4а,6,7,8а,9,10-Octaaza-4,8-dioxo-3,4,4a,7,8,8а,9,9a,10,10а-decahydroanthracene and High-Energy Performance Characterization of Its Dinitramide Salt
Source: Materials (Basel). 2023 Nov 29;16(23):7437. doi: 10.3390/ma16237437 (PMC10706909; doi:10.3390/ma16237437)
Supplement: Supplementary file 1 [file materials-16-07437-s001.zip › materials-2738756-supplementary.pdf]

Supplementary Materials

A New Approach for the Synthesis of 2.3.4a,6.7.8a,9.10–Octaaza-4.8-Dioxo-3.4.4a,7.8.8a,9.9a,10.10a-Decahydroanthracene and High-energy Performance Characterization of Its Dinitramide Salt

Vera S. Glukhacheva[1,*], Sergey G. Il'yasov,[1] Dmitri S. Il'yasov[1], Egor E. Zhukov[1], Ilia V. Eltsov[2], Andrey A. Nefedov[2,3]

[1] Institute for Problems of Chemical and Energetic Technologies, Siberian Branch of the Russian Academy of Sciences (IPCET SB RAS), Biysk 659322, Russia

[2] Department of Natural Sciences, Novosibirsk State University, Novosibirsk 630090, Russia

[3] Novosibirsk Institute of Organic Chemistry, Siberian Branch of the Russian Academy of Sciences (NIOCh SB RAS), Novosibirsk 630090, Russia

**Correspondence. E-mail: vera2878@mail.ru*

Table of Contents

UV spectra of **2, 3** and **4**	S2
IR spectra of **2, 3, 4** and **ADN**	S3
^{1}H and ^{13}C NMR spectra of **3**, **4** and **ADN**	S5
DSC/TGA of **3** and **4**	S11
MS analysis of **2** and **4**	S13
Effect of compound **4** on the burning rate of the Zr/KNO_3 and $KClO_4$/Al pyrotechnic compositions	S19

Figure S1. UV-vis spectra of **2** in H_2O at room temperature

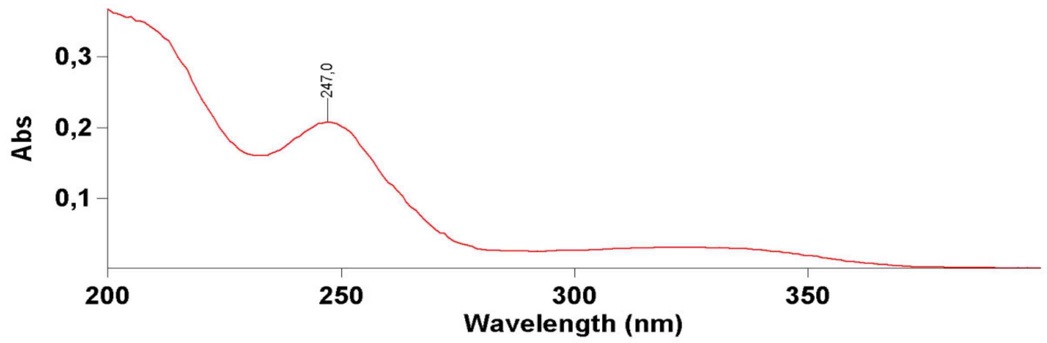

Wavelength (nm)	Abs
247.0	0.219

Figure S2. UV-vis spectra of **3** in H_2O at room temperature

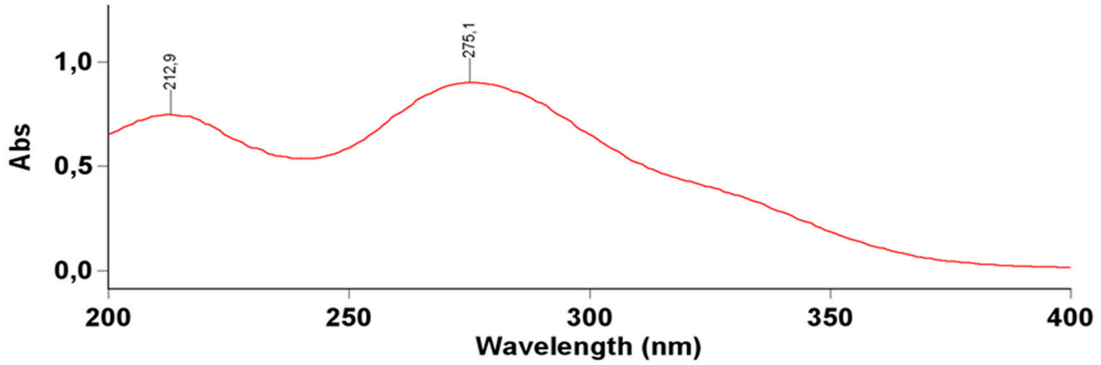

Wavelength (nm)	Abs
275.1	0.900
212.9	0.748

Figure S3. UV-vis spectra of **4** in H_2O at room temperature

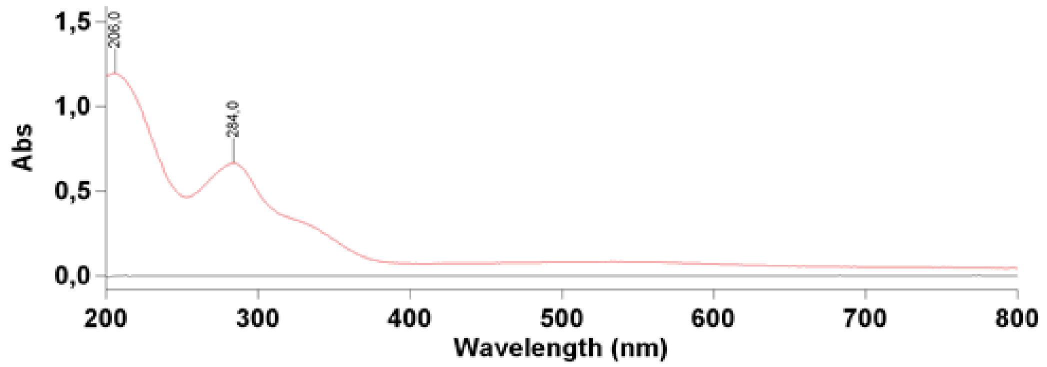

Wavelength (nm)	Abs
284.0	0.664
206.0	1.195

Figure S4. IR spectrum of **2**

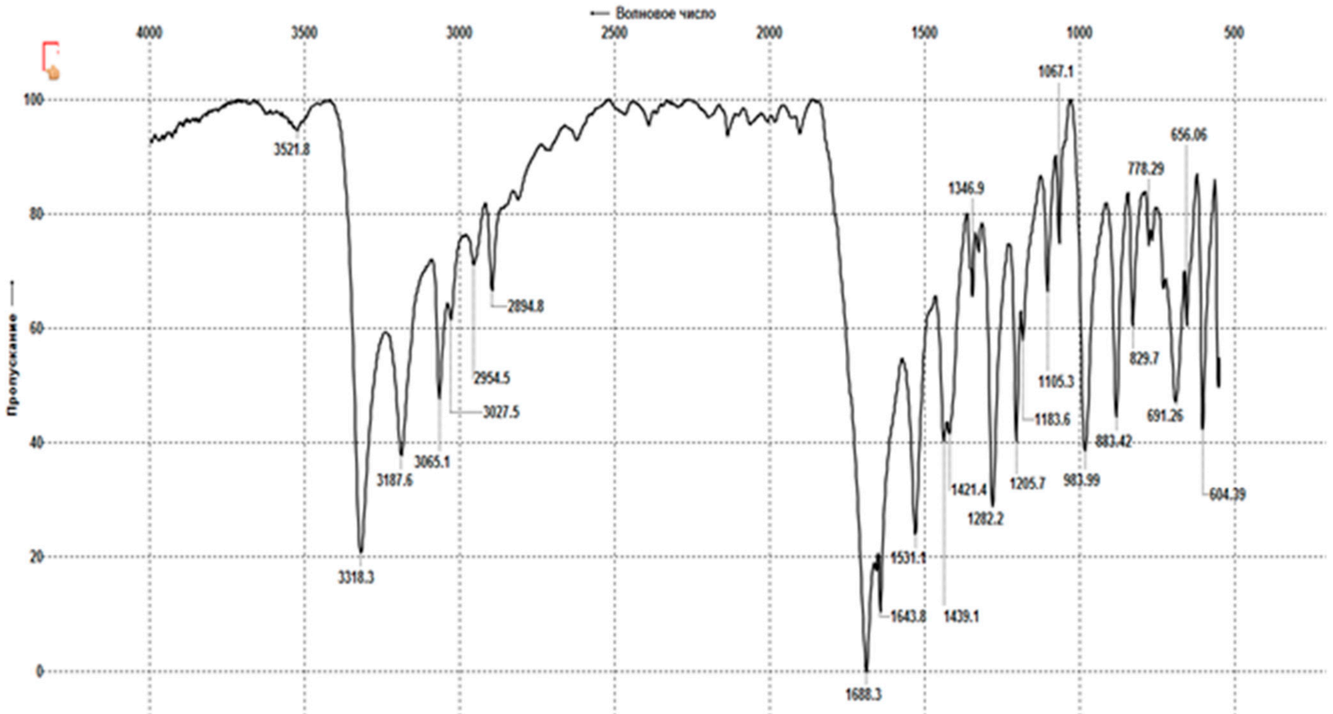

Figure S5. IR spectrum of **3**

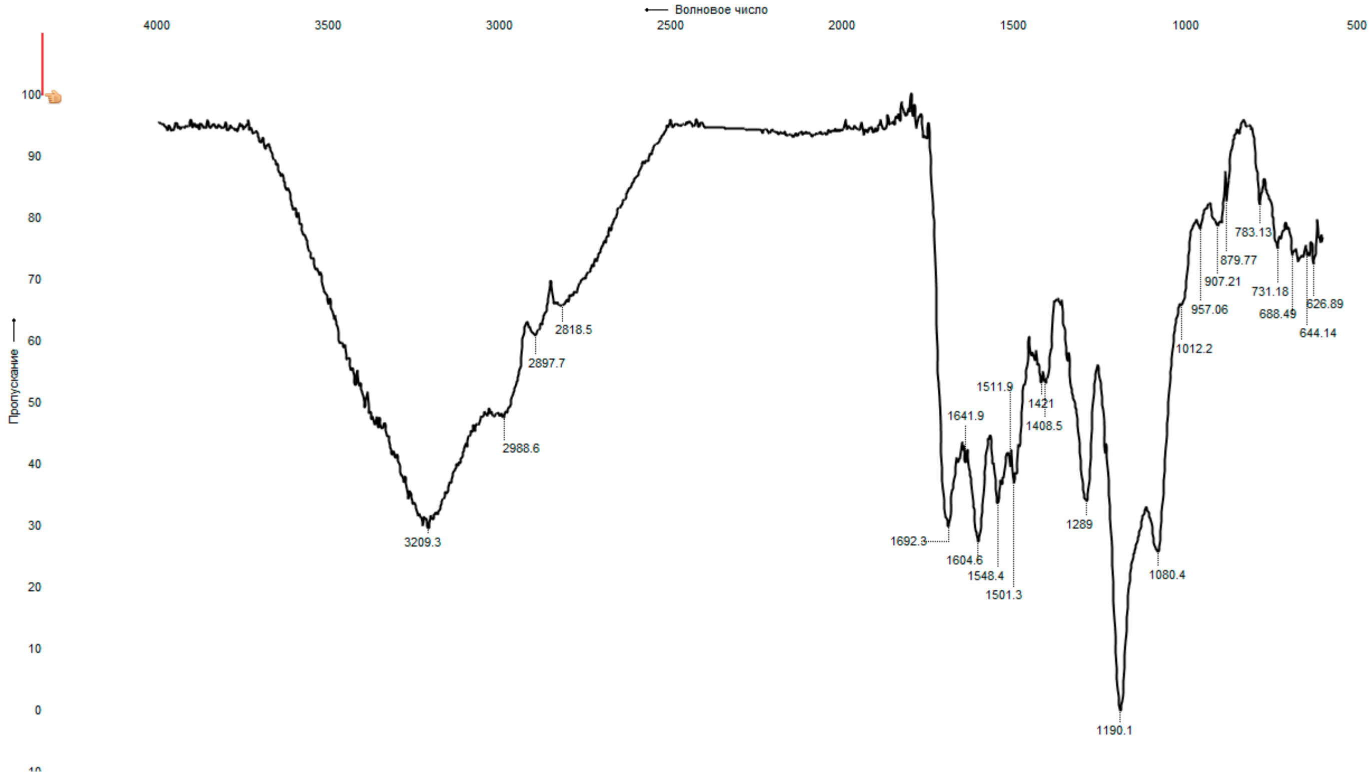

Figure S6. IR spectrum of **4**

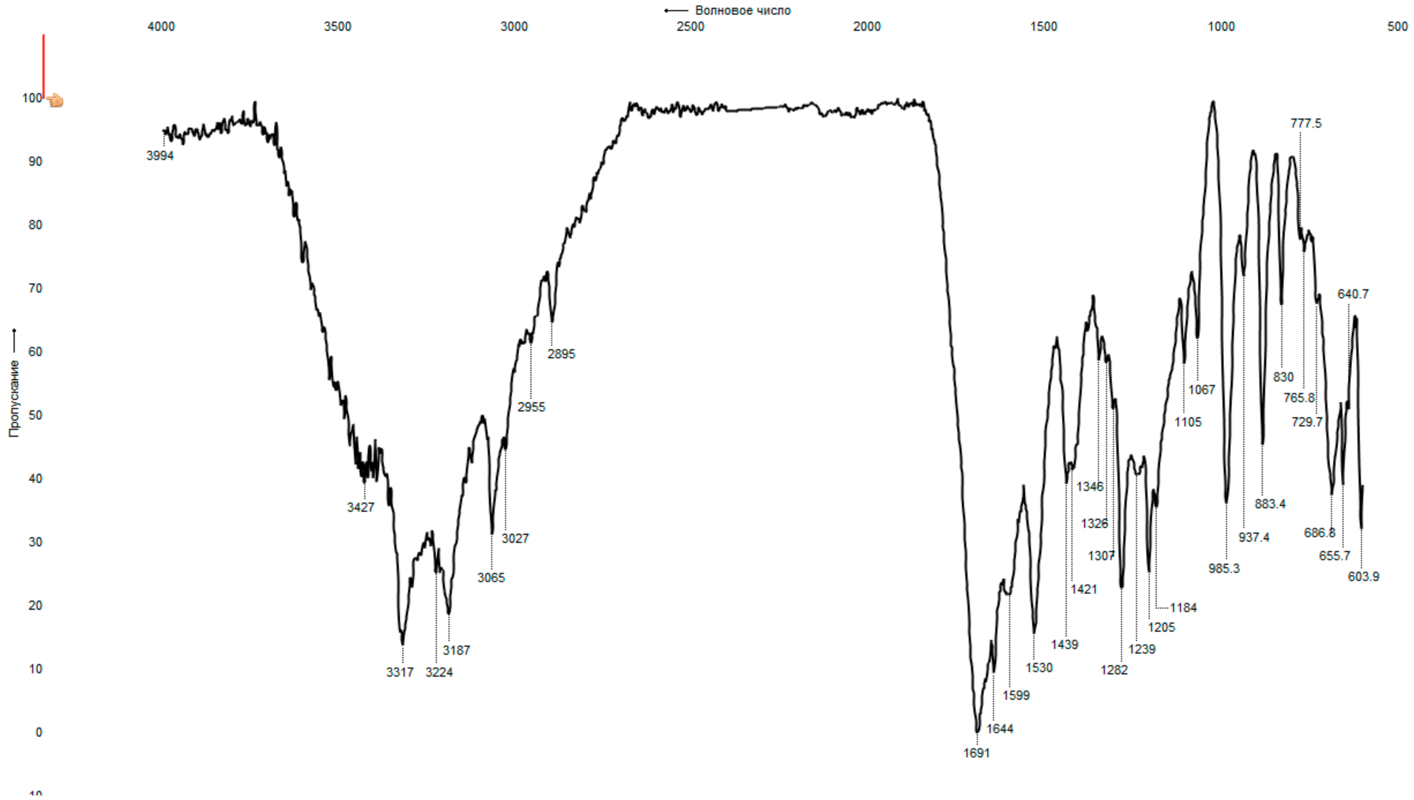

Figure S7. IR spectrum of **ADN**

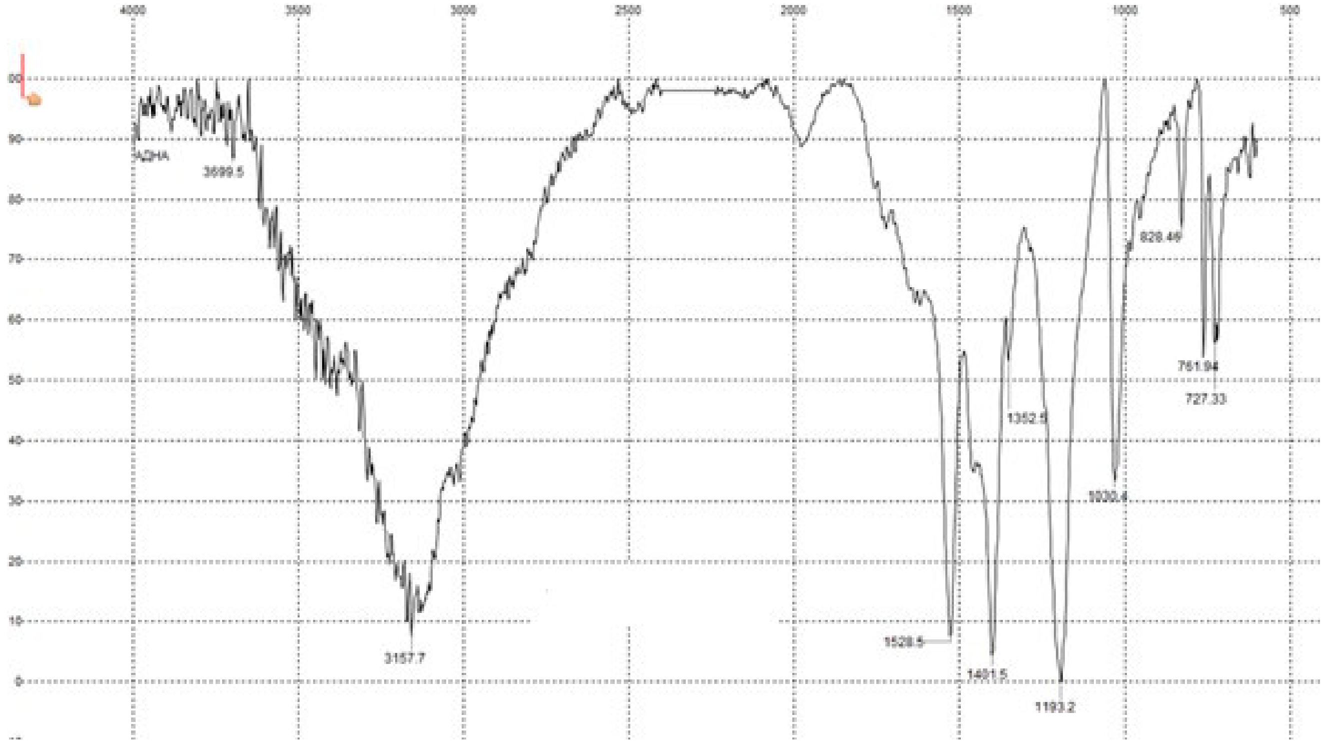

Figure S8. ¹H NMR spectrum of **3** in DMSO, 400.13 MHz (accumulation for 24 h)

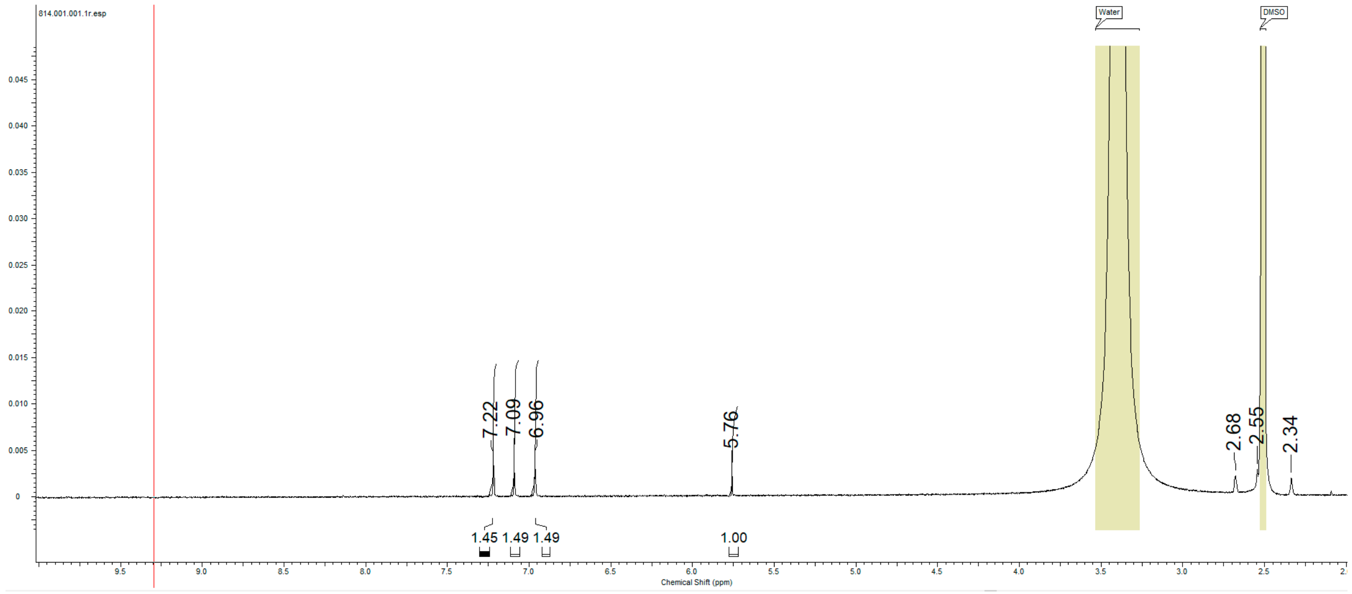

Figure S9. ^{1}H NMR spectrum of **3** in DMSO, 500.03 MHz

An attempt to heat up the sample for 30 min on a water bath at 80–90°C, followed by recording a ^{1}H NMR spectrum under the same conditions, led to the appearance of different signals of 6–11 ppm, which can be assigned both to hydrogen atoms of unsaturated moieties and to hydrogen atoms of NH and NH_2 groups, and so on. A detailed analysis of the resultant mixture proves to be impossible to perform.

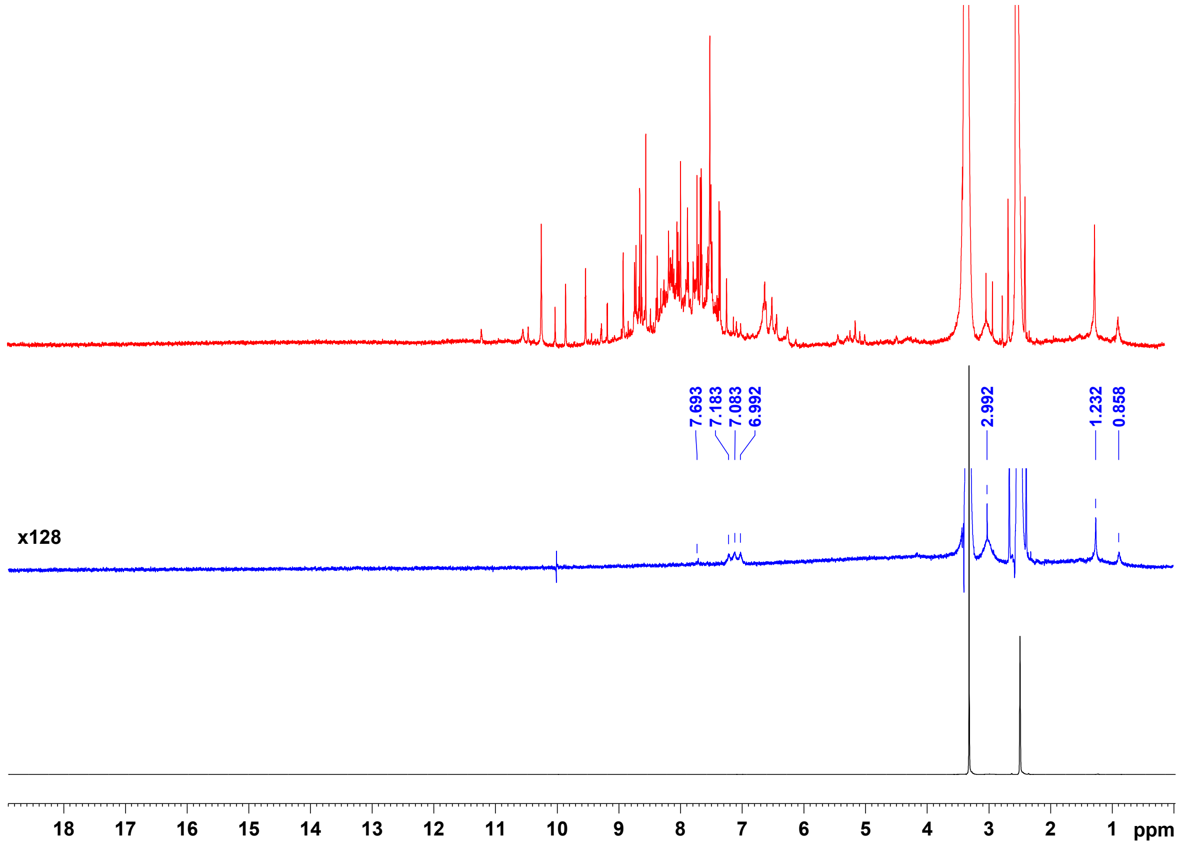

The blue insertion is a spectrum increased by 128 times, and the red insertion is the same sample once heated on a water bath.

Figure S10. ^{1}H NMR spectrum of **3** in D_2O.

Due to the low solubility of the sample in DMSO, an attempt was made to take a spectrum in D_2O and DMF-d_7. The ^{1}H NMR spectrum had quite a small quantity of signals, among which the most intense is a singlet at 8.41 ppm; however, it cannot reliably be asserted that it refers to the azomethine CH=N group: we failed to obtain a correlation of CH at this solubility.

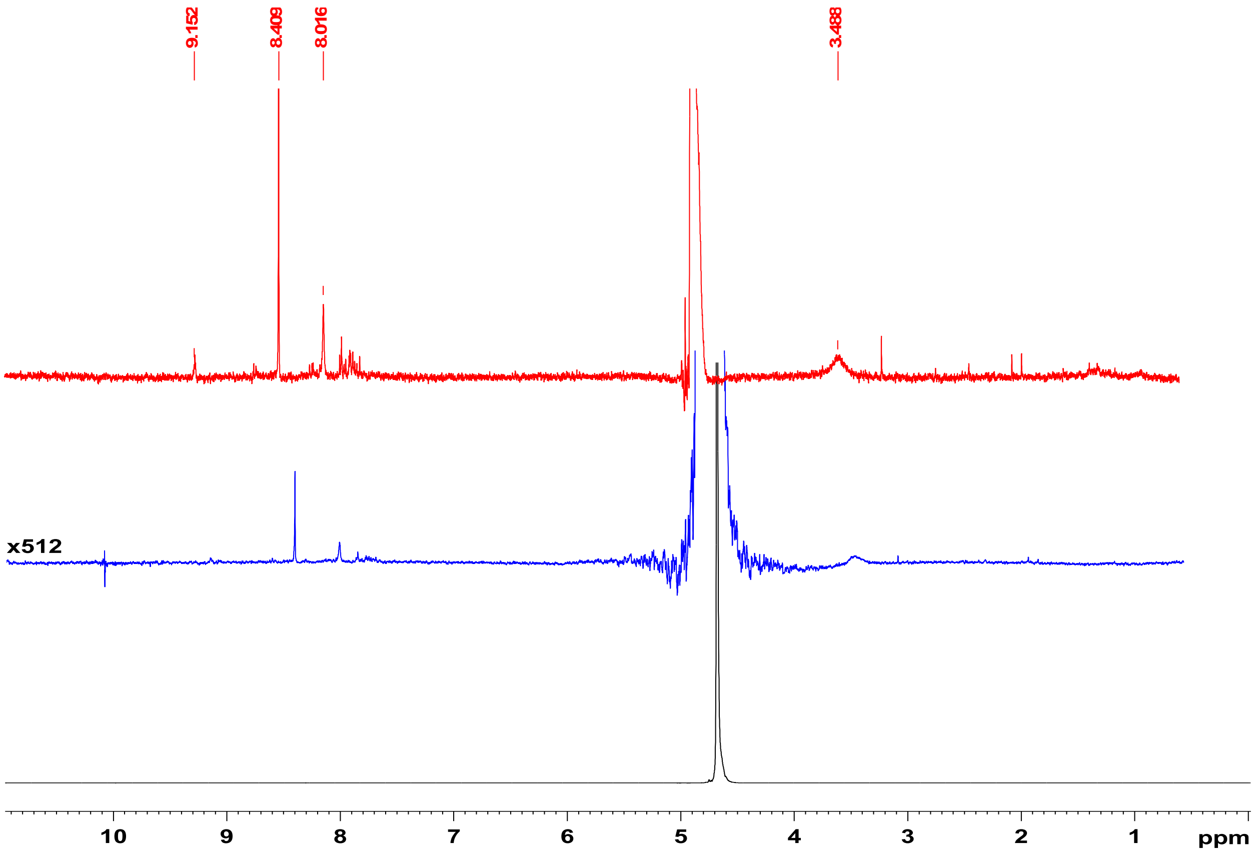

The blue insertion is a spectrum increased by 512 times, and the red insertion is a spectrum obtained via pre-saturation of the water signal.

Figure S11. ^{1}H NMR spectrum of **4** in DMSO, 500.03 MHz

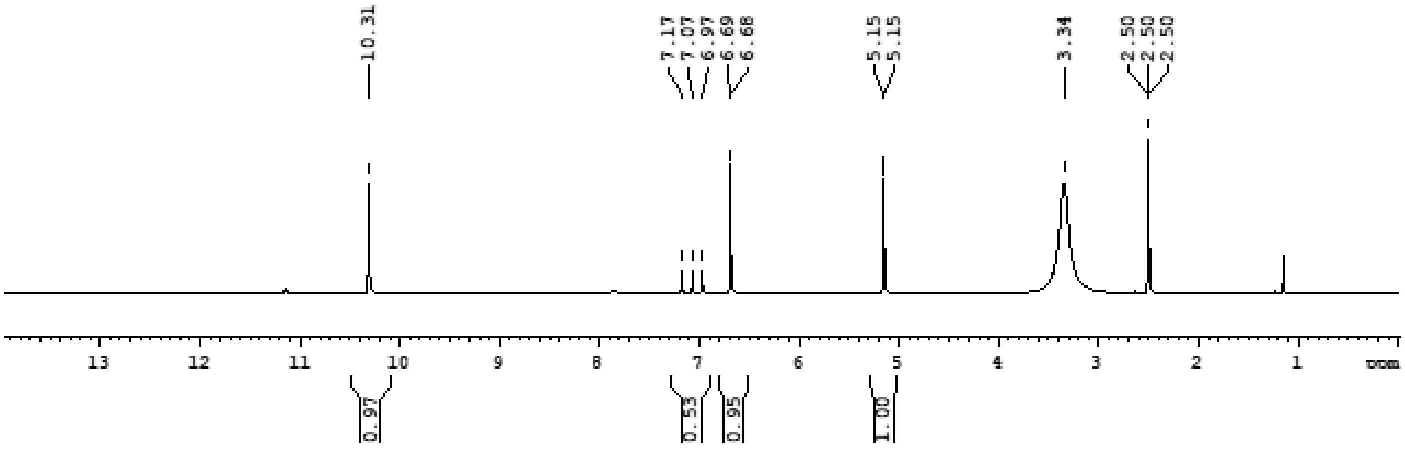

Figure S 12. ^{13}C NMR spectrum of **4** in DMSO

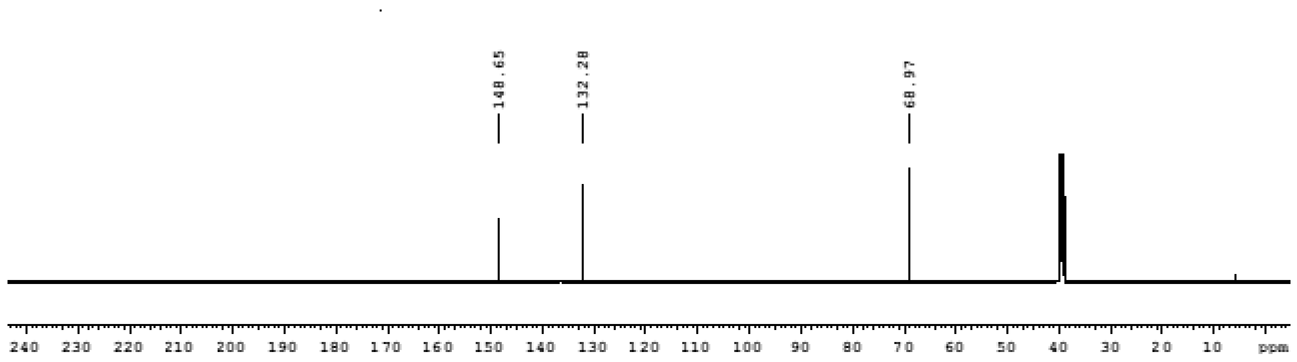

Figure S13. ^{1}H,^{13}C HSQC (red) and ^{1}H,^{13}C HMBC (black) spectrum of **4** in DMSO

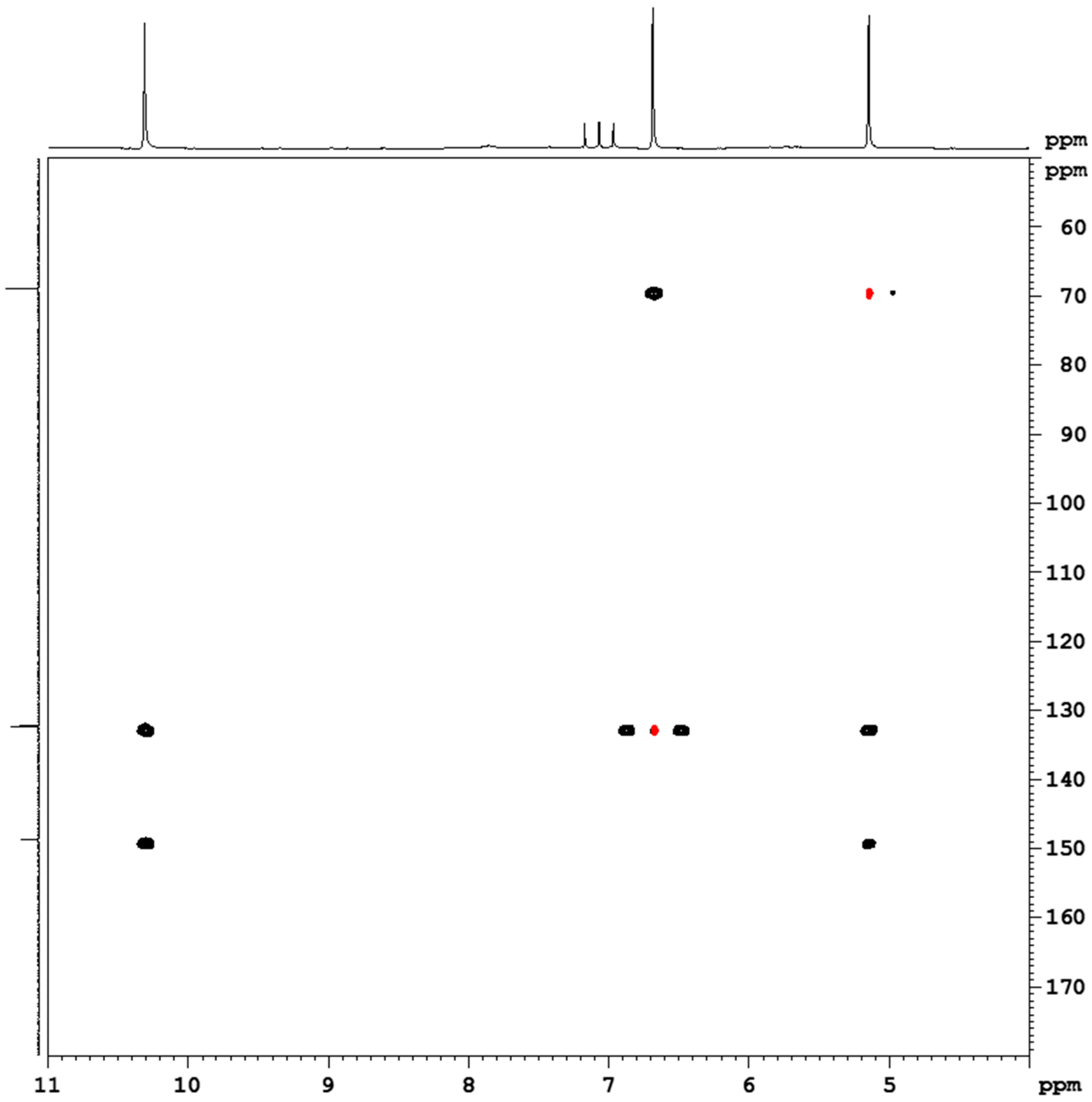

Figure S14. ^{1}H,^{15}N HMBC spectrum of **4** in DMSO

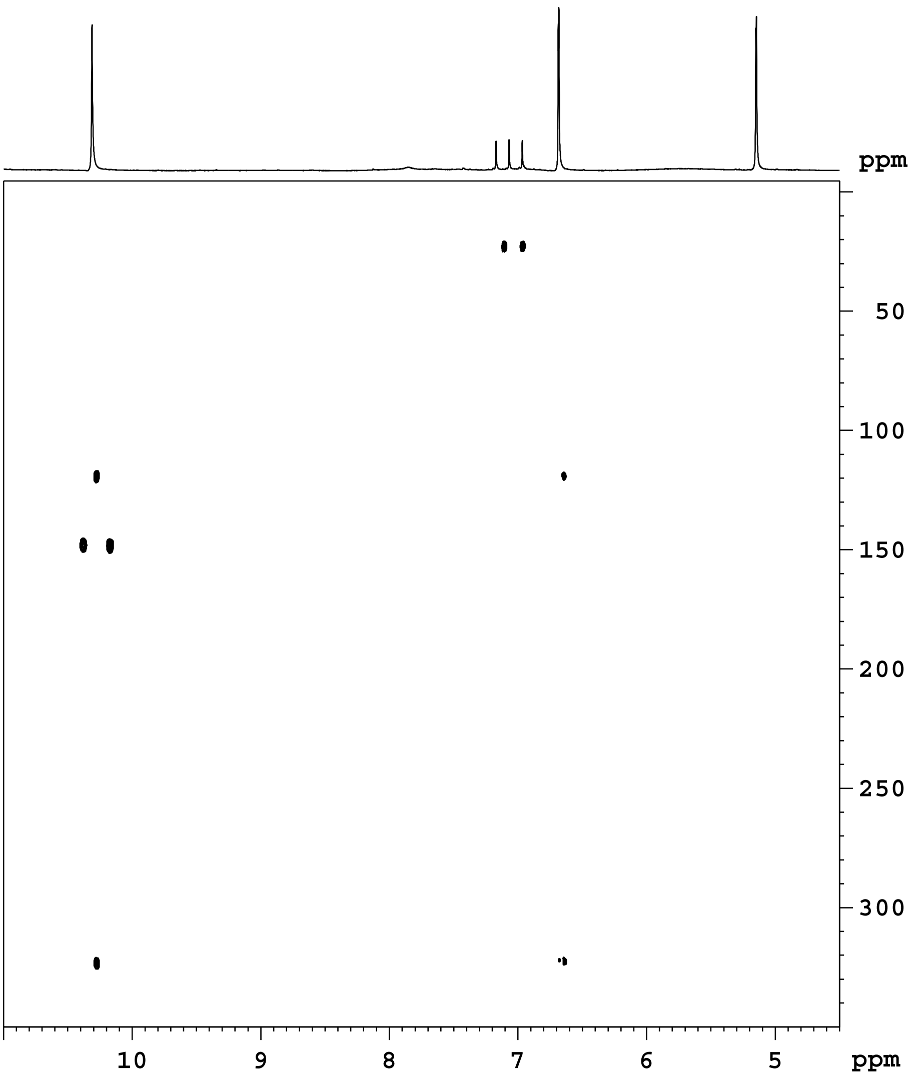

Figure S15. $^{1}H,^{15}N$ HMBC spectrum of **4** in DMSO

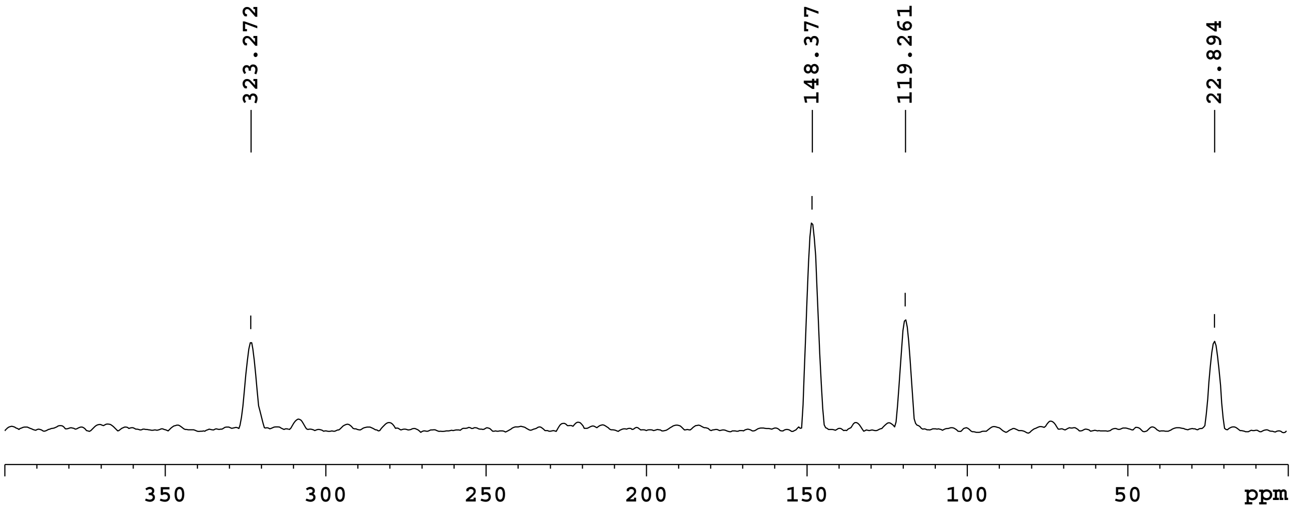

Figure S16. ^{14}N NMR spectrum of **4** in DMSO recorded relative to formamide as reference standard (δ) = 112.5 ppm

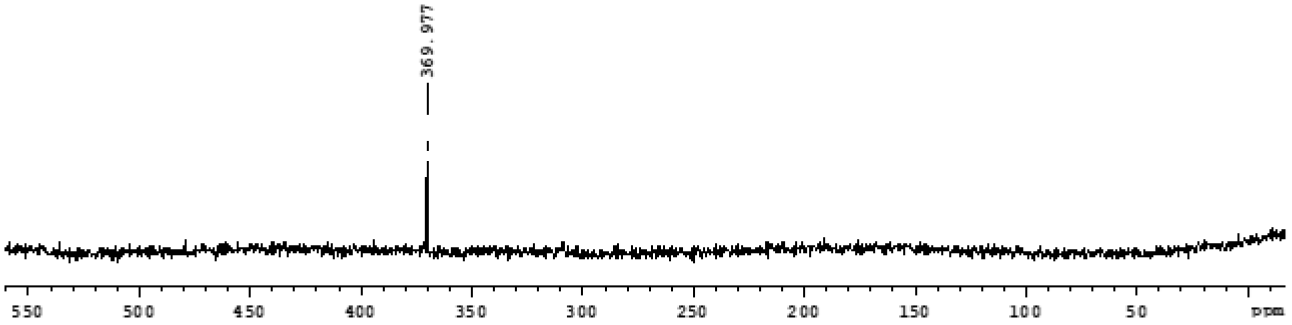

Figure S17. NMR spectrum of **ADN**

ADN was characterized via one-dimensional spectroscopy on ^{1}H and ^{14}N nuclei. The absence of hydrogen atoms in the anion makes analysis via two-dimensional spectroscopy meaningless, and any attempt to obtain a one-dimensional spectrum of ^{15}N using the natural content of the isotope, taking into account the structure of the molecule, is futile. The sample was removed in neat DMSO (19 mg in 0.6 ml) and under acidification conditions using nitric acid (2 drops of concentrated nitric acid).

In the proton spectrum of the sample, a slight impurity signal at 1 ppm, a solvent signal (DMSO, 2.5 ppm), water (3.36 ppm), and a signal from the hydrogen atoms of the ammonium cation were observed. The presence of water in the sample indicates the fact that hydrogen atoms are involved in chemical exchange and appear in the form of a broadened singlet. Acidification of the environment shifts the equilibrium of this process towards stabilization of the NH_4^+ particle, which in turn leads to the manifestation of interaction between the magnetic moments of the ^{1}H and ^{14}N nuclei and transformation of the signal into a triplet with a line ratio of 1:1:1 and a constant ^{1}JNH = 51.2 Hz.

^{1}H-NMR spectra for pure (bottom) and acidified (top) “ADN” sample.

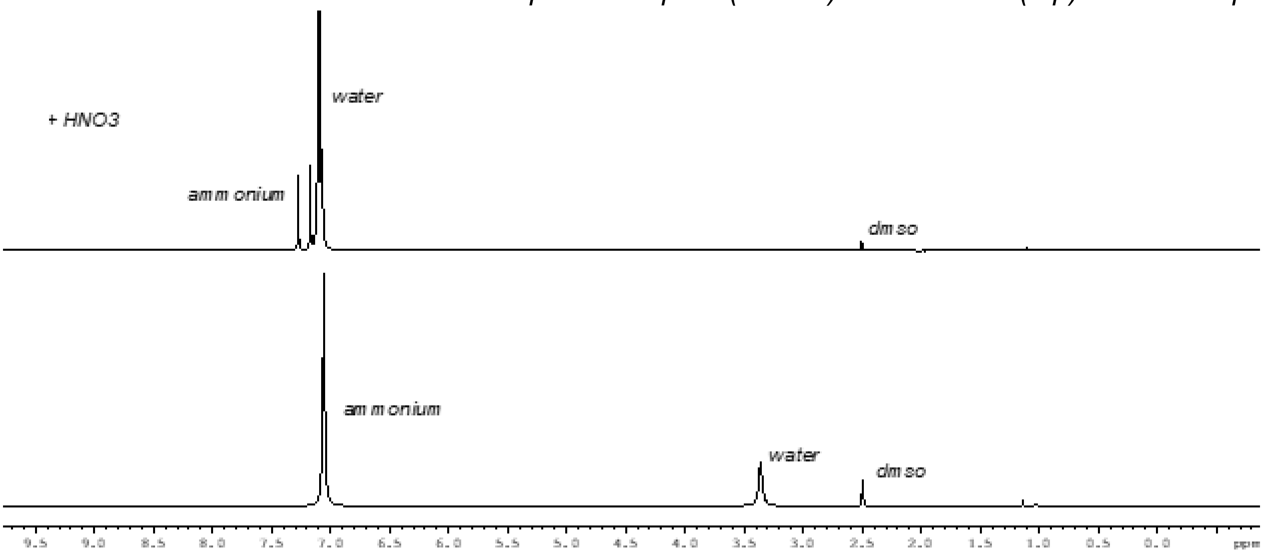

In the spectrum of the ^{14}N nucleus, there are three signals at an integral ratio of 1: 1: 2. The signal at 20.77 ppm corresponds to the nitrogen atom of the ammonium cation and, upon acidification, also exhibits splitting in the form of a quintet with a constant of ^1JNH = 51.2 Hz. The broad signal at 325 ppm corresponds to the central nitrogen atom of the $N(NO_2)_2$ anion. The broadening occurs due to quadrupole interactions with neighboring ^{14}N nuclei. The signal from nitro groups, doubled in intensity, appears at 369 ppm. Acidification of the solution has no effect on the last two signals.

^{14}N-NMR spectra for pure (bottom) and acidified (top) “ADN” sample.

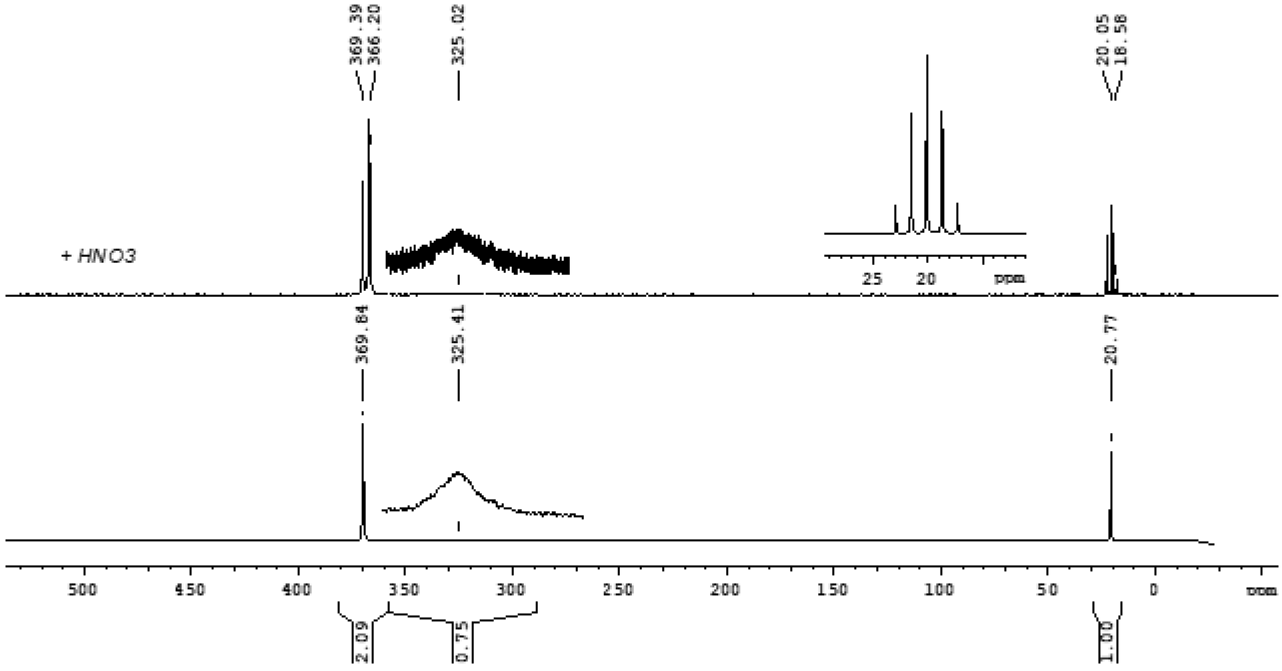

Thus, the following assignment of signals to the connection structure can be performed:

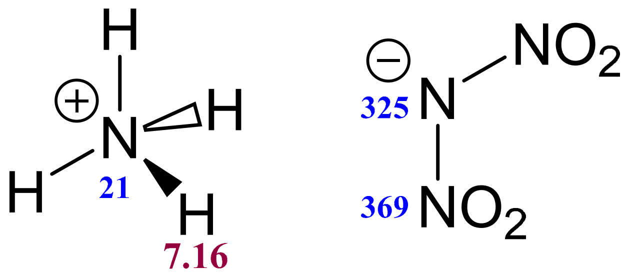

^{1}**H NMR** (500 MHz, DMSO) δ 7.16 (NH_4^+).
^{14}**N NMR** (36 MHz, $HCONH_2$) δ 21 (NH_4^+), 325 (**N**$(NO_2)_2$), 369 (N(**N**$O_2)_2$).

Figure S18. DSC of **3**

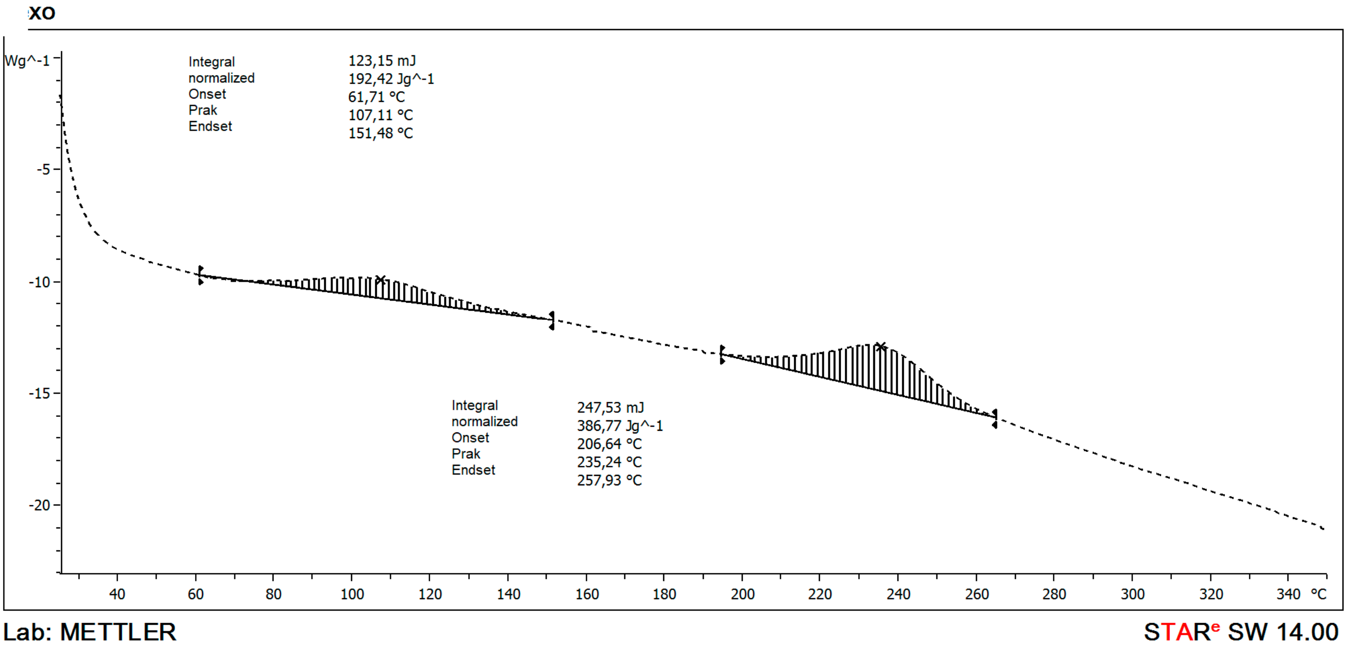

Figure S19. TGA of **3**

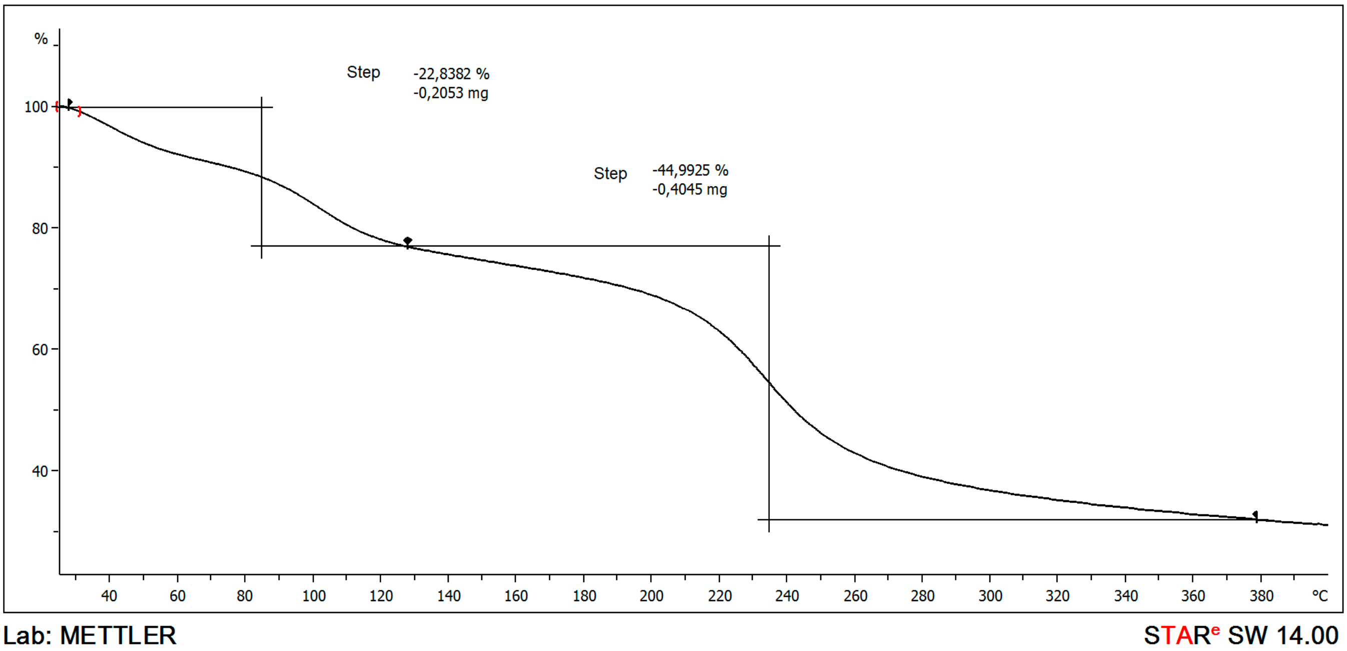

Figure S20. DSC of **4**

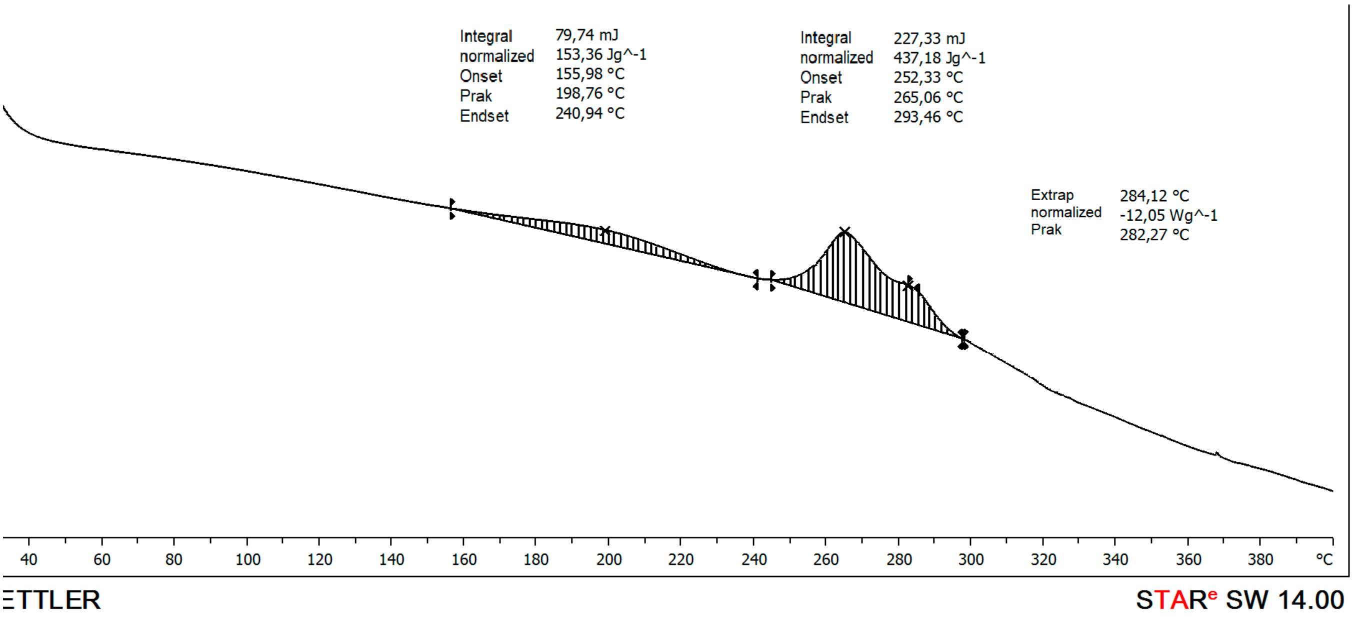

Figure S21. TGA of **4**

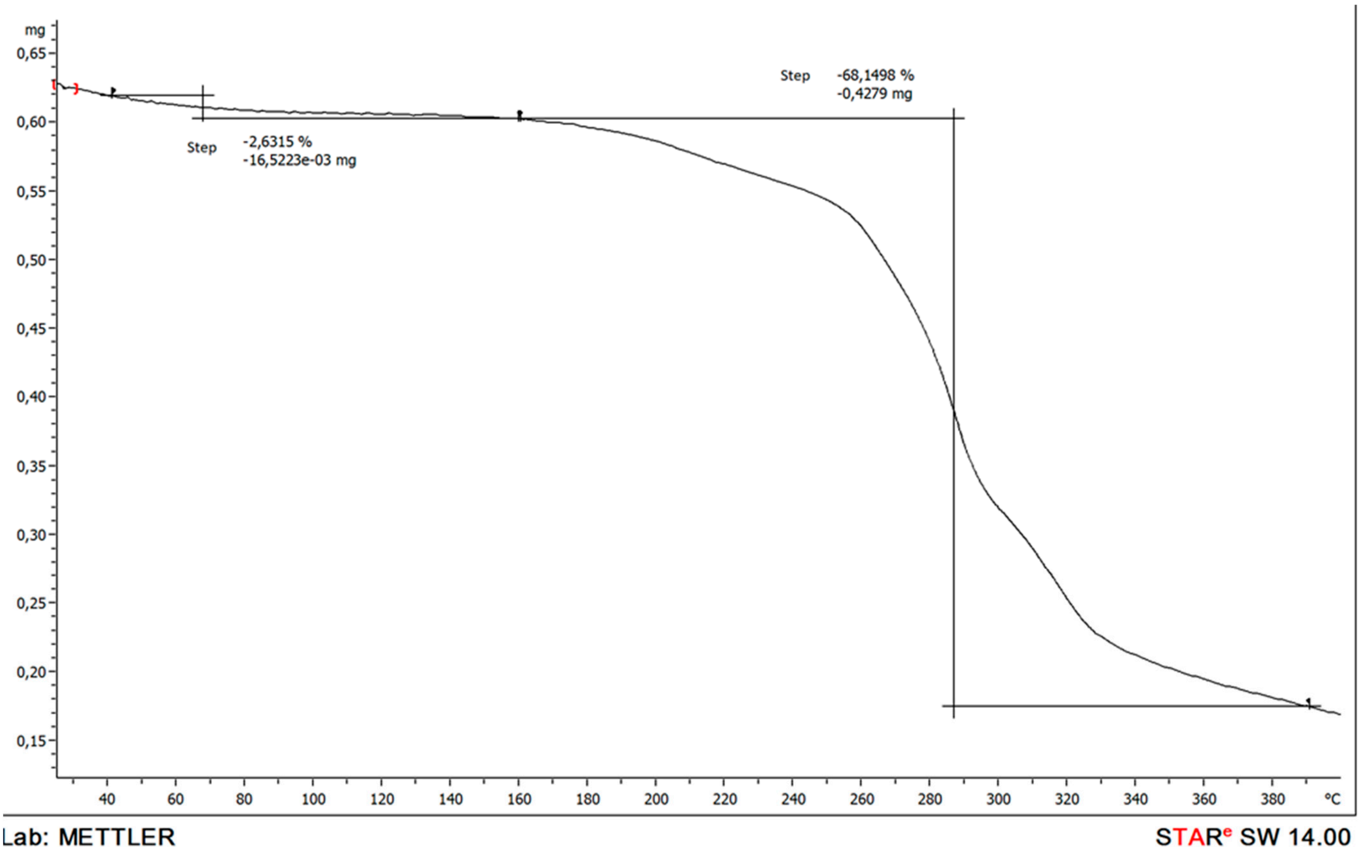

Figure S22. MS analysis of **2**

DFS high-resolution mass-spectrometer

Injection conditions: a direct injection of the sample by using a heated plunger.

T_{source} = 200 ˚C

T_{probe} = 300 ˚C

Electronic Ionization Mass-Spectrometry: HR-MS: Calcd for $C_6H_8N_8O_2$ $[M]^{+\cdot}$ 224.0765; found *m/z* 224.0767. LR-MS, *m/z*, %: 224 (M^+, 16), 113 (100), 112 (16), 98 (59), 56 (12), 44 (13), 43 (17), 42 (15), 40 (13).

Graphic representation of the mass-spectrum:

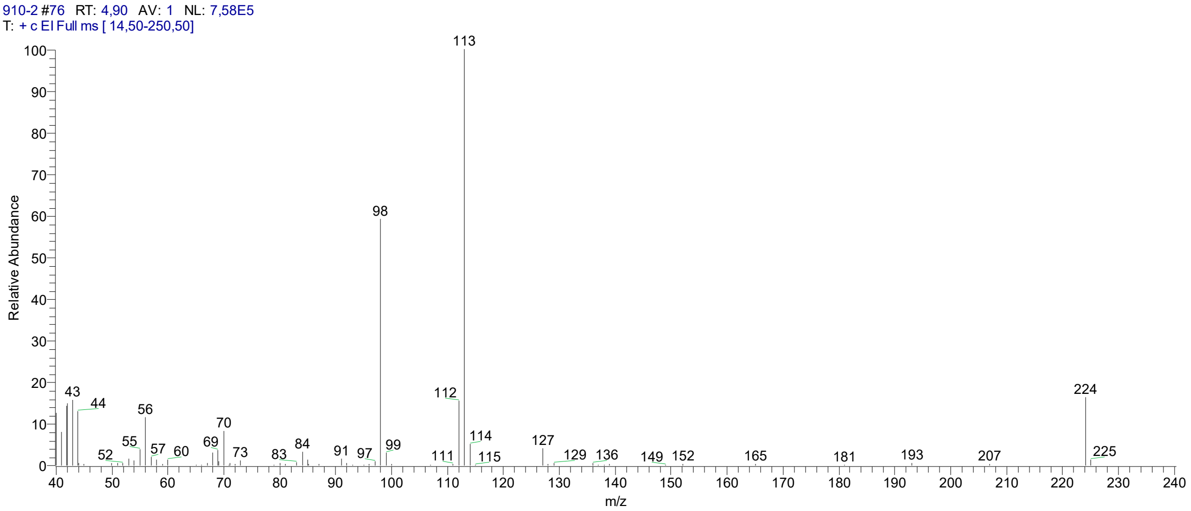

Numerical representation of the mass spectrum:

m/z	Intensity	Relative, %
**40**	**95838.0**	**12.65**
41	60373.0	7.97
**42**	**113617.0**	**14.99**
**43**	**118568.0**	**15.65**
**44**	**98391.0**	**12.98**
45	2490.0	0.33
50	3425.0	0.45
51	4483.0	0.59
52	4639.0	0.61
53	11925.0	1.57
54	7939.0	1.05
55	29299.0	3.87
**56**	**88241.0**	**11.64**
57	14385.0	1.90
58	10088.0	1.33
59	2771.0	0.37
60	10586.0	1.40
65	1494.0	0.20
66	1276.0	0.17
67	4421.0	0.58

68	22978.0	3.03
69	27524.0	3.63
70	63020.0	8.32
71	3829.0	0.51
72	1681.0	0.22
73	8469.0	1.12
79	1152.0	0.15
80	4110.0	0.54
81	2428.0	0.32
83	6165.0	0.81
84	25096.0	3.31
85	9776.0	1.29
87	1774.0	0.23
91	11115.0	1.47
92	4452.0	0.59
93	934.0	0.12
95	1556.0	0.21
96	2584.0	0.34
97	6974.0	0.92
**98**	**449612.0**	**59.33**
99	23165.0	3.06
100	2802.0	0.37
107	560.0	0.07
111	2459.0	0.32
**112**	**117852.0**	**15.55**
**113**	**757865.0**	**100.00**
114	38422.0	5.07
115	1463.0	0.19
127	31292.0	4.13
128	2241.0	0.30
129	4172.0	0.55
136	3580.0	0.47
137	1120.0	0.15
138	1338.0	0.18
139	2210.0	0.29
149	1401.0	0.18
152	1805.0	0.24
165	2553.0	0.34
181	1401.0	0.18
193	3487.0	0.46
207	2086.0	0.28
**224**	**123799.0**	**16.34**
225	10150.0	1.34

Calculation of elemental compositions

Calculated: *m*/*z*=224.0765 ($(C_6H_8N_8O_2)^{+\cdot}$)

Measured: *m*/*z*=224.0767

Elemental composition		RDB	Error (ppm)
1	$C_6 H_8 O_2 N_8$	7.0	1.012

Range of number values of atoms in elements:

Isotope	min	max
**12 C**	**4**	**10**
**1 H**	**4**	**20**
**16 O**	**0**	**6**
**14 N**	**0**	**10**

$(M)^{+\cdot}$ —a positively charged odd-electron molecular radical ion (radical cation).

Figure S23. MS analysis of **4**

DFS high-resolution mass-spectrometer

Injection conditions: a direct injection of the sample by using a heated plunger.

T_{source} = 200 °C

T_{probe} = 280 °C

Electronic Ionization Mass-Spectrometry: HR-MS: Calculated for $C_6H_8N_8O_2$ $[M]^{+\cdot}$ 224.0765; found *m*/*z* 224.0763.

Graphical representation of mass-spectrum:

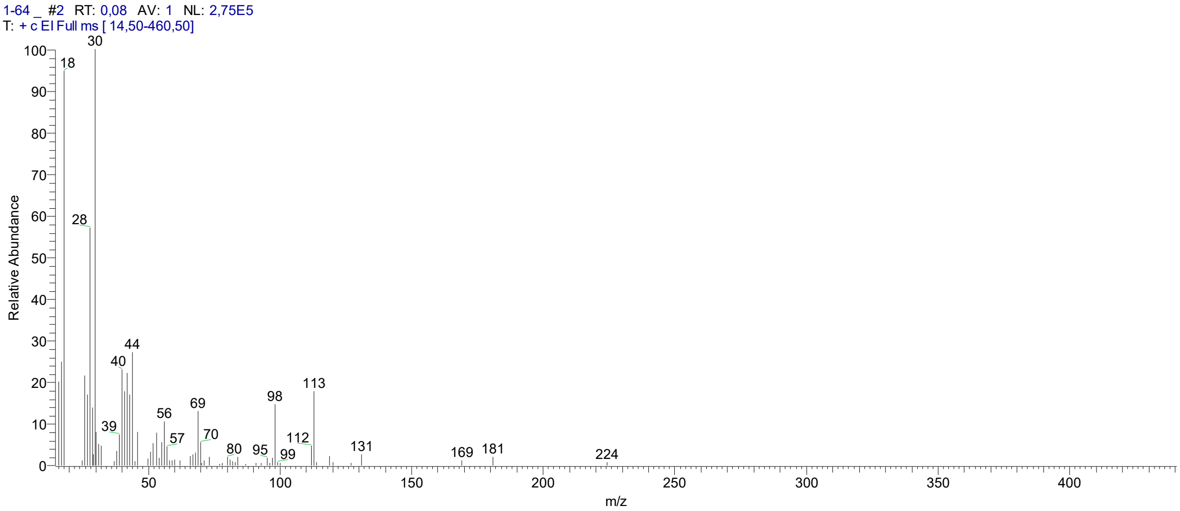

Graphical representation of mass-spectrum (enlarged)

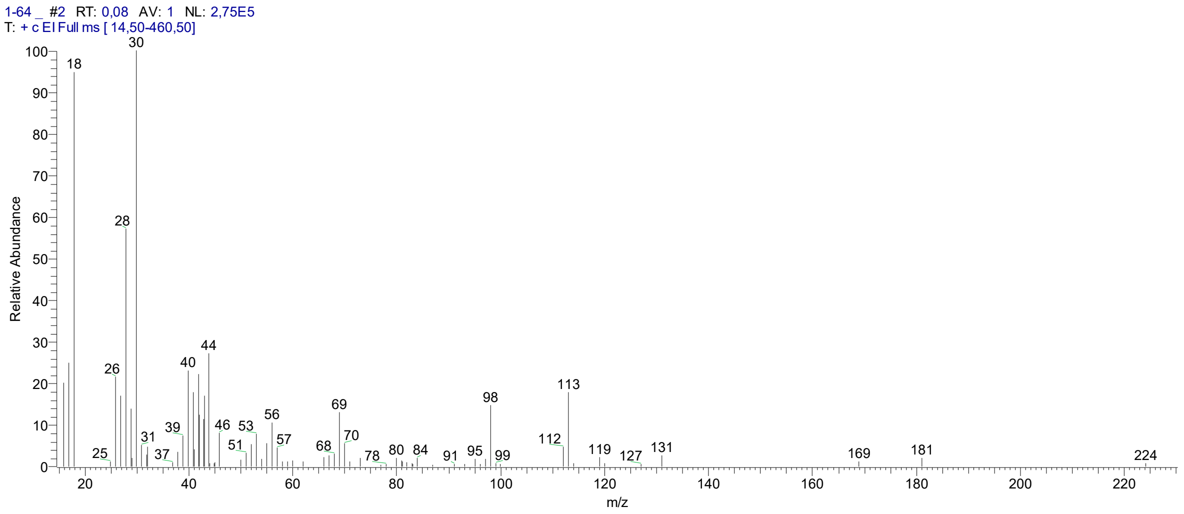

There are ions from nitro groups with *m/z* =46 (NO_2) and *m/z*=30 (NO) and intramolecular redox products with *m/z*=18 (H_2O) and *m/z*=28 (N_2).

Numerical representation of mass-spectrum:

m/z	**Intensity**	**Relative, %**
16	54971.0	20.00
17	68299.0	24.85
18	260700.0	94.86
25	3150.0	1.15
26	59402.0	21.61
27	46871.0	17.05
28	157438.0	57.29
29	38044.0	13.84
30	274824.0	100.00
31	13985.0	5.09
32	12946.0	4.71
37	2388.0	0.87
38	9381.0	3.41
39	20389.0	7.42
40	63314.0	23.04
41	48740.0	17.73
42	61237.0	22.28
43	46767.0	17.02
44	74807.0	27.22
45	2734.0	0.99
46	22154.0	8.06
50	4500.0	1.64
51	9138.0	3.33
52	14642.0	5.33
53	21254.0	7.73
54	4846.0	1.76

55	15127.0	5.50
56	29009.0	10.56
57	12185.0	4.43
58	3288.0	1.20
59	3219.0	1.17
60	3877.0	1.41
62	3011.0	1.10
66	5815.0	2.12
67	7096.0	2.58
68	8031.0	2.92
69	35932.0	13.07
70	15335.0	5.58
71	2977.0	1.08
73	5261.0	1.91
77	761.0	0.28
78	1176.0	0.43
80	5469.0	1.99
81	3634.0	1.32
82	2561.0	0.93
83	1938.0	0.71
84	5296.0	1.93
87	1038.0	0.38
91	1661.0	0.60
93	1523.0	0.55
95	4880.0	1.78
96	1661.0	0.60
97	4811.0	1.75
98	40571.0	14.76
99	1869.0	0.68
100	1315.0	0.48
112	12635.0	4.60
113	49086.0	17.86
114	2111.0	0.77
119	5919.0	2.15
120	2215.0	0.81
127	1592.0	0.58
131	7442.0	2.71
169	3253.0	1.18
181	5504.0	2.00
224	2007.0	0.73

Calculation of elemental compositions:

Calculated: *m*/*z*=438.0699 $(C_6H_{10}N_{14}O_{10})^{+\cdot}$ — not detected.

Measured: *m*/*z*=224.0763

**Elemental composition**		**RDB**	**Error (ppm)**
1	$C_6 H_8 O_2 N_8$	7.0	-0.773
2	$C_7 H_{14} O_7 N_1$	1.5	-0.797

Calcd for $(C_6H_8N_8O_2)^{+\cdot}$ *m*/*z*=224.0765

Range of number values of atoms in elements:

**Isotope**	**min**	**max**
**12 C**	**4**	**10**
**1 H**	**4**	**20**
**16 O**	**0**	**12**
**14 N**	**0**	**20**

$(M)^{+\cdot}$ — a positively charged odd-electron molecular radical ion (radical cation).

Figure S24. Effect of compound **4** on the burning rate of the $KClO_4$/Al pyrotechnic composition

The following formulation was used as the model pyrotechnic composition: 70 % potassium perchlorate (ACS grade, technical specifications No. 6-09-3801-76) and 30 % aluminum (ASD-4 brand, technical specifications No. 48-5-226-87).

Compound **4** was used as the modifier.

no additive							
l, mm	t, s	u, mm/s	avr.	disp.	SD	CI	half-interval
4.94	1.42	3.47	3.27	0.11	0.33	0.22	0.11
5.14	1.53	3.35					
5.00	1.65	3.04					
5.14	1.52	3.38					
5.20	1.64	3.17					
5.10	1.45	3.51					
5.00	1.85	2.71					

5.00	1.67	2.99					
5.14	1.34	3.83					

0.5% **4** additive							
l, mm	t, s	u, mm/s	avr.	disp.	SD	CI	half-interval
5.00	1.74	2.87	2.98	0.11	0.34	0.22	0.11
5.26	1.58	3.33					
5.00	1.84	2.71					
5.10	1.97	2.59					
5.22	1.68	3.10					
5.24	1.82	2.87					
5.00	1.75	2.86					
4.98	1.36	3.67					
4.98	1.78	2.79					

1% **4** additive							
l, mm	t, s	u, mm/s	avr.	disp.	SD	CI	half-interval
5.12	1.81	2.83	2.87	0.22	0.47	0.34	0.17
5.18	1.95	2.65					
5.16	1.85	2.80					
5.10	1.79	2.86					
5.22	1.42	3.67					
5.20	2.00	2.60					
5.10	1.32	3.86					
5.14	1.89	2.72					
5.24	2.21	2.37					

1.5% **4** additive							
l, mm	t, s	u, mm/s	avr.	disp.	SD	CI	half-interval
5.00	2.11	2.37	2.49	0.12	0.35	0.23	0.12

5.10	2.21	2.30					
5.10	1.51	3.37					
5.12	2.15	2.38					
5.22	2.00	2.62					
5.00	2.02	2.48					
5.24	2.38	2.20					
5.22	2.32	2.25					
5.10	2.08	2.45					

Figure S25. Effect of compound **4** on the burning rate of the Zr/KNO_3 pyrotechnic composition

The following formulation was used as the model pyrotechnic composition: 52 % zirconium (PCZr-1 brand (powdered calciothermic Zr, technical specifications No. 48-4-234-84) and 48 % potassium nitrate (ACS grade, GOST R 4217-77).

Compound **4** was used as the modifier.

no additive						
l ,mm	t ,s	u, mm/s	avr.	disp.	SD	half-interval
4.42	0.11864	37.26	34.10	4.0282	2.0070	1.3908
4.36	0.11967	36.43				
4.22	0.12108	34.85				
4.38	0.12851	34.08				
4.18	0.12901	32.40				
4.24	0.13071	32.44				
4.42	0.14007	31.56				
4.36	0.12910	33.77				
4.44	0.12745	34.84				

0.5% **4** additive							
l, mm	t, s	u, mm/s	avr.	disp.	SD	CI	half-interval
4.42	0.14213	31.10	29.76	3.6703	1.9158	1.4192	0.7096

4.36	0.14892	29.28					
4.4	0.15031	29.27					
4.32	0.14517	29.76					
4.42	0.14852	29.76					
4.4	0.14552	30.24					
4.32	0.16523	26.15					
4.28	0.14813	28.89					
4.42	0.13244	33.37					

1% **4** additive							
l, mm	t, s	u, mm/s	avr.	disp.	SD	CI	half-interval
4.46	0.16520	27.00	27.30	2.6521	1.6285	1.0640	0.5320
4.24	0.15479	27.39					
4.22	0.16289	25.91					
4.2	0.16851	24.92					
4.32	0.14751	29.29					
4.24	0.15849	26.75					
4.2	0.14223	29.53					
4.4	0.15221	28.91					
4.28	0.16472	25.98					

1.5% **4** additive							
l, mm	t, d	u, mm/s	avr.	disp.	SD	CI	half-interval
4.42	0.16952	26.07	23.79	3.9259	1.9814	1.5854	0.7927
4.24	0.18315	23.15					
4.2	0.20642	20.35					
4.22	0.17852	23.64					
4.32	0.17211	25.10					
4.4	0.18010	24.43					
4.22	0.16958	24.89					
4.44	0.16848	26.35					
4.2	0.18112	23.19					
